# Influenza virus polymerase subunits co-evolve to ensure proper levels of dimerization of the heterotrimer

**DOI:** 10.1371/journal.ppat.1008034

**Published:** 2019-10-03

**Authors:** Kuang-Yu Chen, Emmanuel Dos Santos Afonso, Vincent Enouf, Catherine Isel, Nadia Naffakh

**Affiliations:** 1 Unité de Génétique Moléculaire des Virus à ARN, Institut Pasteur, UMR 3569 CNRS, Paris, France; 2 Unité de Génétique Moléculaire des Virus à ARN, Université Paris Diderot, Sorbonne Paris Cité, Paris, France; 3 Unité de Génétique Moléculaire des Virus à ARN, Centre National de Référence des Virus des Infections Respiratoires, Institut Pasteur, Paris, France; 4 Pasteur International Bioresources network (PIBnet), Plateforme de Microbiologie Mutualisée (P2M), Institut Pasteur, Paris, France; Emory University School of Medicine, UNITED STATES

## Abstract

The influenza A virus RNA-dependent RNA polymerase complex consists in three subunits, PB2, PB1 and PA, that perform transcription and replication of the viral genome through very distinct mechanisms. Biochemical and structural studies have revealed that the polymerase can adopt multiple conformations and form oligomers. However so far it remained unclear whether the available oligomeric crystal structures represent a functional state of the polymerase. Here we gained new insights into this question, by investigating the incompatibility between non-cognate subunits of influenza polymerase brought together through genetic reassortment. We observed that a 7:1 reassortant virus whose PB2 segment derives from the A/WSN/33 (WSN) virus in an otherwise A/PR/8/34 (PR8) backbone is attenuated, despite a 97% identity between the PR8-PB2 and WSN-PB2 proteins. Independent serial passages led to the selection of phenotypic revertants bearing distinct second-site mutations on PA, PB1 and/or PB2. The constellation of mutations present on one revertant virus was studied extensively using reverse genetics and cell-based reconstitution of the viral polymerase. The PA-E349K mutation appeared to play a major role in correcting the initial defect in replication (cRNA -> vRNA) of the PR8xWSN-PB2 reassortant. Strikingly the PA-E349K mutation, and also the PB2-G74R and PB1-K577G mutations present on other revertants, are located at a dimerization interface of the polymerase. All three restore wild-type-like polymerase activity in a minigenome assay while decreasing the level of polymerase dimerization. Overall, our data show that the polymerase subunits co-evolve to ensure not only optimal inter-subunit interactions within the heterotrimer, but also proper levels of dimerization of the heterotrimer which appears to be essential for efficient viral RNA replication. Our findings point to influenza polymerase dimerization as a feature that is controlled by a complex interplay of genetic determinants, can restrict genetic reassortment, and could become a target for antiviral drug development.

## Author summary

Influenza viruses, because of their yearly recurrence and the occasional emergence of pandemics, represent a worldwide major public health threat. Enhancing the fundamental knowledge on the influenza RNA-dependent RNA-polymerase, a PB1-PB2-PA heterotrimer which ensures transcription and replication of the viral genome, is essential to reach the goal of better prevention and treatment of the disease. Here we gained new insights into the viral polymerase function by investigating the incompatibility between polymerase subunits derived from distinct parental influenza viruses and brought together through genetic reassortment. Our data show that the polymerase subunits co-evolve to ensure not only optimal inter-subunit cooperation within the heterotrimer, but also proper levels of dimerization of the heterotrimer which appears to be essential for efficient viral RNA replication. Our findings point to influenza polymerase dimerization as a feature that can restrict genetic reassortment, a major evolutionary mechanism for influenza viruses, and could become an attractive target for antiviral drug development.

## Introduction

Influenza A viruses (IAV) are respiratory pathogens that belong to the family of *Orthomyxoviridae* (for a review, see [1]). They represent a worldwide and major public health threat due to their ease of transmission, their ability to cause moderate to severe respiratory symptoms, their persistence in animal reservoirs and their genetic variability. Influenza A viruses have a segmented genome composed of eight single-stranded, negative-sense viral RNAs (vRNAs) that range in length from 0.9 to 2.3 kb and together encode ten major and several auxiliary proteins. Each vRNA is associated with nucleoprotein (NP) oligomers and with a copy of the viral heterotrimeric RNA-dependent RNA polymerase (FluPol) consisting of the PB2, PB1 and PA subunits, to form viral ribonucleoproteins (vRNPs). Fully infectious progeny virions contain a complete set of eight distinct vRNPs that are selectively co-packaged through segment-specific *cis*-acting packaging signals.

Following viral infection, vRNPs are imported into the nucleus of the host cell, where the FluPol performs transcription and replication of the viral genome, through very distinct mechanisms [2]. Transcription into messenger RNAs (mRNAs) involves a cap-snatching step by which 5’-capped oligonucleotides, derived from nascent cellular capped RNAs, are used as primers and elongated by the polymerase, and a polyadenylation step. In contrast, replication is primer-independent and proceeds through complementary RNAs (cRNAs) which serve as templates for amplification of the vRNAs. Three-dimensional structures of RNA-free [3–4], vRNA promoter-bound [4–6] or cRNA promoter-bound FluPol [5, 6] have been recently resolved. They reveal that the heterotrimeric polymerase complex can undergo major conformational rearrangements, which likely contribute to its capacity to perform vRNA->mRNA, vRNA->cRNA or cRNA->vRNA synthesis. In addition to tight and dynamic interactions between the subunits that form the heterotrimer, there is evidence for interactions between distinct heterotrimers. Indeed, FluPol oligomers have been detected upon transient expression of PB2, PB1 and PA in cultured cells [7] or FluPol purification [4, 8, 9]. Moreover, structural and functional studies of IAV negative- and positive-sense RNPs have led to models of genome replication (cRNA -> vRNA) that rely on a *trans*-acting [10] or a *trans*-activating FluPol [11]. Still, the mechanistic of how the PB2, PB1 and PA subunits cooperate to ensure the function and regulation of FluPol’s different conformational states remains largely unknown.

Studies on reassortant IAV viruses can provide insights into functional interactions between FluPol subunits. Indeed the segmented nature of IAV genome allows, during mixed infections, the production of reassortant viruses that harbor combinations of genomic segments derived from different parental viruses, in a process named genetic reassortment. Reassortment between co-circulating human IAVs is shaping the evolution of seasonal IAVs, while genetic reassortment between animal and human IAVs can facilitate the emergence of antigenically novel and potentially pandemic viruses [12–14]. The diversity of IAV reassortment is limited by RNA- and protein-based incompatibilities between co-infecting viruses (for a review see [15]). There is evidence that nucleotide differences in the genomic packaging signals can result in suboptimal interactions between the heterologous vRNAs and therefore limit the diversity of IAVs produced after a co-infection event [16], and the recent elucidation of the structure of the IAV genome has provided additional demonstration of the role of vRNA-vRNA interactions in co-segregation of viral segments during reassortment [17]. Suboptimal physical and/or functional interactions among heterologous viral proteins manifest when newly formed progeny viruses infect a new cell, leading to an inefficient or abortive infection. The most documented examples of protein incompatibility involve the balance between the receptor-binding and receptor-destroying activities of the hemagglutinin (HA) and neuraminidase (NA) surface glycoproteins, e.g. [18, 19], and the interplay between the three FluPol subunits, e.g. [20, 21]. Several observations point towards a genetic linkage between the PB2 and PA subunits. For instance, a mismatch between PB2 and PA can impair the assembly or activity of reassortant FluPols in a minigenome assay [20–22], and cognate PB2-PA segments tend to co-segregate in panels of reassortant viruses produced upon experimental, e.g. [21, 23] or natural, e.g. [24, 25] co-infections. Notably, the reassortant viruses responsible for the 1957, 1968 and 2009 pandemics contained PB2 and PA segments that were phylogenetically related to the same lineage whereas PB1 had a distinct origin [14]. However cases of genetic linkage between PB1 and PB2 have also been documented [26, 27].

Here we found that a 7:1 reassortant virus whose PB2 segment derives from the A/WSN/33 (WSN) virus in an otherwise A/PR/8/34 (PR8) backbone is strongly attenuated, despite a 97% identity between the PR8-PB2 and WSN-PB2 proteins. Several independent revertant viruses were obtained after serial passages, showing different sets of second-site mutations that we submitted to genetic and functional analysis. Using cell-based assays to express the FluPol we found that second-site mutations on PA, PB1 or PB2 which contribute to the phenotypic reversion increase FluPol activity while they decrease the level of FluPol dimerization. By revealing how the three FluPol subunits co-evolve to ensure proper levels of dimerization for optimal viral replication, our data provide new insights into the conformational regulation of FluPol activity.

## Results

### Reversion of an attenuated PR8xWSN-PB2 reassortant virus

**Fig 1:**
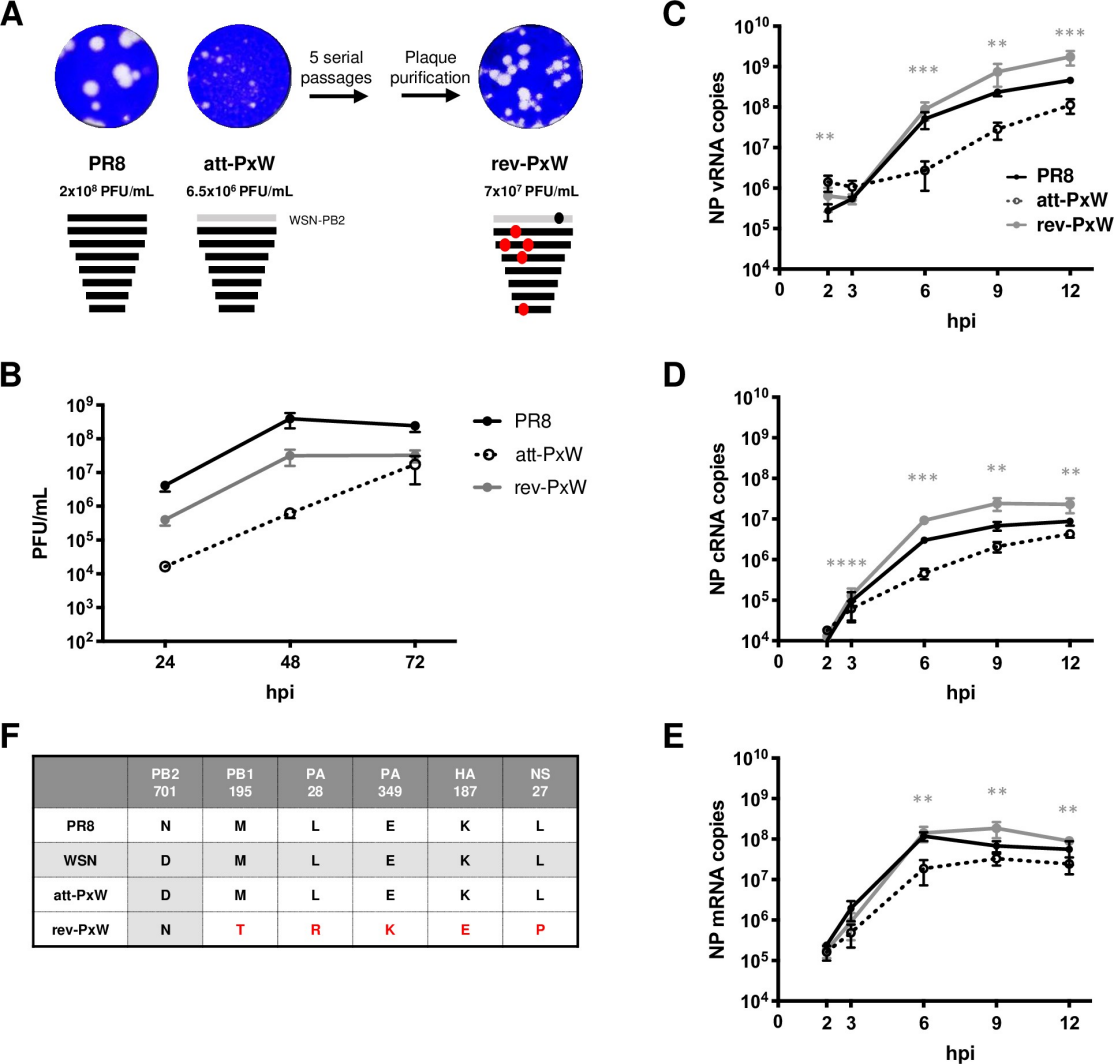
Phenotypic and genotypic characterization of the wild-type, attenuated and revertant viruses. **(A)** Plaque phenotype and titers of the wild-type PR8 virus, attenuated PR8xWSN-PB2 reassortant virus (att-PxW) and revertant virus (rev-PxW) isolated upon 5 serial passages of att-PxW. Following reverse genetics (PR8, att-PxW) or plaque purification (rev-PxW), the viruses were amplified once and titrated on MDCK cells. Crystal violet staining of infected cell monolayers is shown. **(B)** Growth kinetics under multi-cycle conditions. A549 cells were infected at a m.o.i. of 0.001 with the indicated viruses. At the indicated times post-infection, viral titers were determined by plaque assay on MDCK cells. The results are shown as the mean ± SD of three independent experiments. **(C-E)** Levels of NP v-, c- and mRNAs under single cycle conditions. A549 cells were infected at a m.o.i. of 5 and the levels of NP vRNAs (**C**), cRNAs (**D**) and mRNAs (**E**) were determined at the indicated times post-infection by strand-specific RT-qPCR. The copy numbers are shown as the mean ± SD of four independent experiments in duplicate. Significance was assessed using Student’s paired t-test after log transformation (**p ≤ 0.01; *** p ≤ 0.001; **** p ≤ 0.0001, rev-PxW compared to att-PxW). **(F)** Whole genome sequence. The three amplified viral stocks were subjected to vRNA extraction, RT-PCR of the eight genomic segments and next-generation sequencing. The amino acid changes observed in the rev-PxW virus compared to the initial att-PxW virus are indicated.

The A/WSN/33 (WSN) and A/Puerto Rico/8/34 (PR8) laboratory strains both derive from early human A(H1N1) IAVs and have been extensively passaged. Here we used well-characterized recombinant WSN [28] and PR8 [29] viruses whose FluPol subunits show 97 to 98% amino acid identity. Still, a 7:1 reassortant virus in which only the PB2 segment derives from WSN whereas the remaining segments derive from PR8 (named thereafter att-PxW virus) was found to be attenuated: it grew to lower titers (6.5x10^6^ PFU/mL) compared to the wild-type PR8 virus produced in the same reverse genetics experiment (2x10^8^ PFU/mL) and formed small plaques (**Fig 1A**).

The att-PxW virus was submitted to serial passages at a low multiplicity of infection (m.o.i.) on MDCK cells, until a virus forming large plaques was detected at passage 5 and was cloned by two plaque purification rounds. This phenotypic revertant virus, named rev-PxW, grew at high titers (7x10^7^ PFU/mL, **Fig 1A**). To compare the viral growth kinetics of the wild-type PR8 virus with the att-PxW and the rev-PxW viruses (see **Fig 1B**), human pulmonary A549 cells were infected at low m.o.i. and the infectious titers in the supernatant were determined 24, 48 and 72 hours post-infection (hpi). While the growth kinetic of the att-PxW virus was considerably slowed down, with titers decreased by more than two log at 24 and 48 hpi compared to wild-type PR8, the rev-PxW grew significantly better albeit not reaching wild-type levels (**Fig 1B**), in accordance with its titer and plaque phenotype (**Fig 1A**). The rev-PxW virus was also used, in parallel with the att-PxW reassortant and the parental PR8 viruses, to measure the levels of NP vRNAs, cRNAs and mRNAs by strand-specific RT-qPCR, in single-cycle infections of A549 cells, from 2 to 12 hpi. All three types of viral RNAs were significantly higher with the rev-PxW compared to the att-PxW virus, reaching or even exceeding the wild-type PR8 levels (**Fig 1C–1E**).

Sequencing of the revertant’s full genome by Next Generation Sequencing (NGS) revealed 6 amino acid changes compared to the initial att-PxW virus, including 4 changes on the FluPol segments (**Fig 1F**). One of these, the PB2-D701N substitution, corresponded to a reversion from the WSN-PB2 towards the PR8-PB2 sequence at position 701; the other three changes (PB1-M195T, PA-L28R, and PA-E349K) occurred at residues of PR8-PB1 and -PA that were conserved between PR8 and WSN (**Fig 1F**). These extragenic changes seemed likely to contribute to the phenotypic reversion, as (i) upon serial passages and plaque-purification of the parental PR8 virus, no amino acid changes in the FluPol genes were observed and (ii) the substitutions observed in the revertant virus were unusual. Indeed, residues PB1-195T, PA-28R and PA-349K were found in only 0.16%, 0.02% and 0.2%, respectively, of large PA and PB1 sequence datasets representative of > 3000 human IAVs (see Materials and Methods section and **S1 Table**). The two other changes (NS1-L27P and HA-K187E,) were less likely to compensate for the presence of a heterologous PB2 subunit.

### Reversion is due to second-site mutations on PA

**Fig 2:**
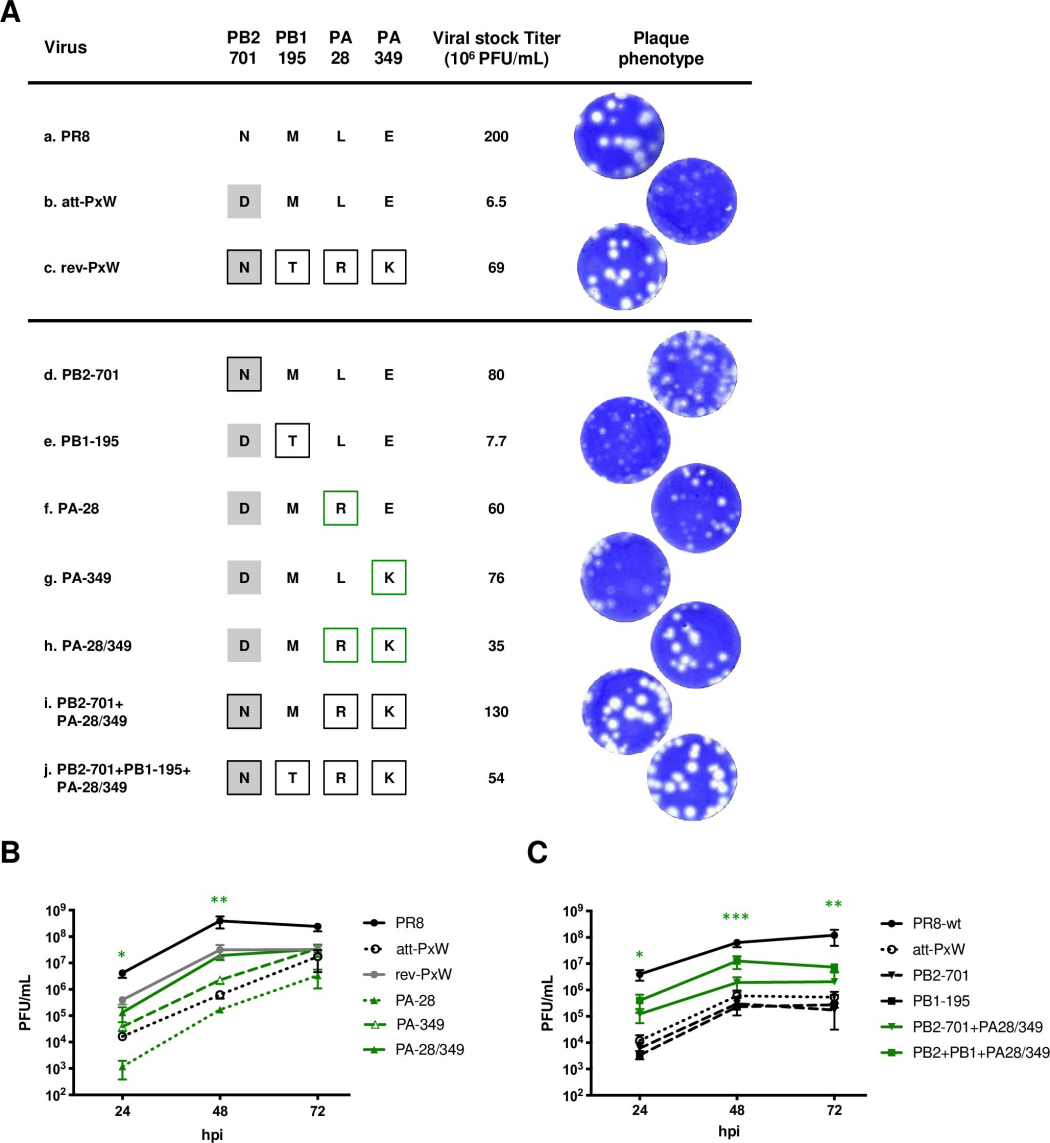
Contribution of FluPol amino acid changes to the titer and plaque phenotype of the rev-PxW virus. **(A)** Titers and plaque phenotype of recombinant viruses bearing one or several reversion mutations in the att-PxW genetic background, as follows: PB2-D701N (**d**), PB1-M195T (**e**), PA-L28R (**f**), PA-E349K (**g**), PA-L28R/E349K (**h**) PB2-D701N+PA-L28R/E349K (**i**) and PB2-D701N+PB1-M195T+PA-L28R/E349K (**j**). Recombinant PR8 (**a**) and att-PxW (**b**) viruses were rescued in parallel. Following one round of amplification on MDCK cells, the titers and plaque phenotypes were compared to that of the rev-PxW virus (**c**). Grey squares represent a WSN-PB2 background. Residues that are mutated compared to the att-PxW virus are framed. Green frames correspond to the green color code used in the following figures. **(B-C)** Growth kinetics under multi-cycle conditions. A549 cells were infected at a m.o.i. of 0.001 with the indicated viruses. At the indicated times post-infection, viral titers were determined by plaque assay on MDCK cells. The results are shown as the mean ± SD of three independent experiments. The data shown for PR8, att-PxW and rev-PxW viruses in (B) are the same as in Fig 1B. The significance was tested with a Student’s paired t-test after log transformation (*p≤0.05; **p≤0.01; *** p ≤ 0.001, PA-28/349 compared to att-PxW in (B), PB2+PB1+PA28/349 compared to att-PxW in (C).

The contribution of each of the PB2-D701N, PB1-M195T, PA-L28R, and PA-E349K mutations to the phenotypic reversion was analyzed using reverse genetics (below and in the figures they are referred to as mutations PB2-701, PB1-195, PA-28 and PA-349). Recombinant viruses with isolated or combined mutations in the att-PxW background were produced and their genotype was confirmed by whole genome NGS sequencing. The virus with the full-set of four mutations showed a viral titer and plaque phenotype close to the revertant virus, as expected (**Fig 2A**, **j** compared to **c**). So did the viruses with the three (PB2-701 + PA-28 + PA-349) or two (PA-28 + PA-349) mutations (**Fig 2A**, **h** and **i** compared to **c**). Viruses with only one mutation in PB2 or PA grew to high titers but displayed an intermediate plaque phenotype between the att- and rev-PxW viruses (**Fig 2A**, **d-g** compared to **b** and **c**). The PB1 mutation, either isolated, or in addition to the PB2 and PA mutations, did not impact the plaque phenotype or viral titer (**Fig 2A**, **e** and **j** compared to **b** and **i**, respectively). The viral growth kinetics of the the mutant viruses were compared to those of the PR8 and att-PxW viruses (**Fig 2B and 2C**). While the growth kinetics of viruses with an isolated PA mutation were slowed down (PA-28) or only slightly enhanced (PA-349) compared to the att-PxW virus, the PA-28/349 virus grew significantly better than the att-PxW virus and as efficiently as the rev-PxW revertant (**Fig 2B**, green lines). The PB2-701 and PB1-195 viruses displayed growth kinetics similar to the att-PxW virus (**Fig 2C**, dashed lines). Viruses harbouring the PB2 or PB2 and PB1 reversion mutations combined with the PA-28/349 mutations behaved like the PA28/349 mutant virus, *i.e.* grew better than the att-PxW virus albeit not reaching wild-type levels (**Fig 2C**, green lines).

**Fig 3:**
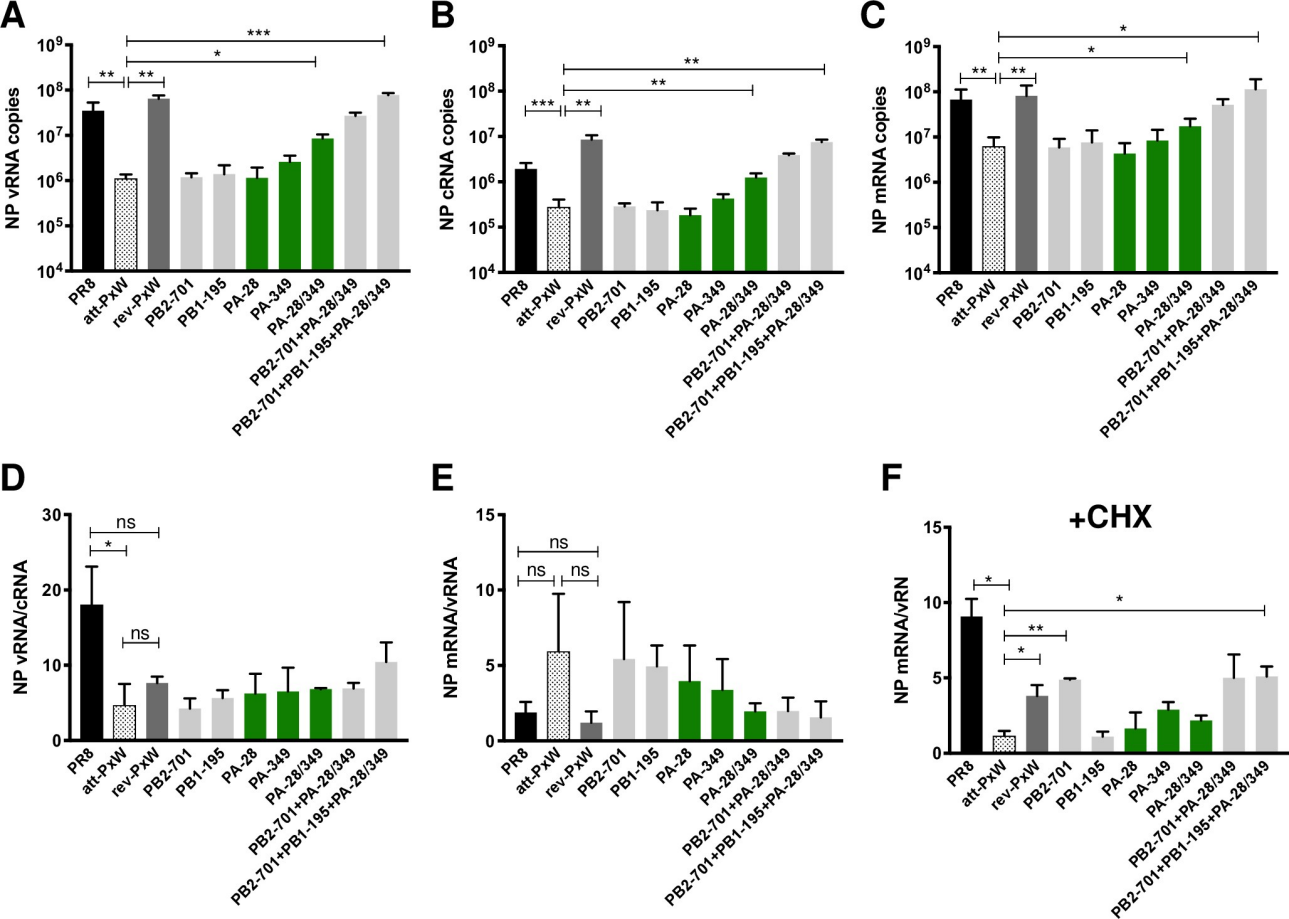
Contribution of FluPol amino acid changes to viral replication capacity. **(A-C)** Levels of NP v-, c- and mRNAs under single cycle conditions. A549 cells were infected at a m.o.i. of 5 and the levels of NP vRNAs (**A**), cRNAs (**B**) and mRNAs (**C**) were determined at 6 hpi by strand-specific RT-qPCR. The copy numbers are shown as the mean ± SD of three independent experiments in duplicate. Significance was assessed using Student’s paired t-test after log transformation (*p ≤ 0.05; **p ≤ 0.01; *** p ≤ 0.001). **(D-E)** The levels of NP v-, c-, and m-RNAs determined at 6 hpi were used to calculate the vRNA/cRNA (**D**) and mRNA/vRNA (**E**) copy number ratios. **(F)** Accumulation of primary NP transcripts in the presence of cycloheximide (CHX). A549 cells were infected as in (A-C) in the presence of 100 μg/mL of CHX *i*.*e*. under conditions of primary transcription. The levels of NP v- and m-RNAs determined at 6 hpi were used to calculate the mRNA/vRNA ratio. The results are shown as the mean ± SD of three (**D-E**) or two (**F**) independent experiments in duplicates. Significance was assessed using Student’s unpaired t-test (*p ≤ 0.05; **p ≤ 0.01; ns: non significant).

The mutant viruses were used for single-cycle infection of A549 cells and the levels of NP vRNAs, cRNAs and mRNAs were determined by strand-specific RT-qPCR at 6 hpi (**Fig 3A–3E**). In agreement with our initial observations (**Fig 1C–1E**), the levels of all three types of viral RNAs were significantly reduced with the att-PxW virus (**Fig 3A–3C**, dotted bars) compared to the PR8 (black bars) or revertant (dark grey bars) viruses. Individual PB2, PB1 or PA mutations had no effect. The two combined PA mutations resulted in significantly increased levels of v-, c- and mRNAs compared to the att-PxW virus. With the addition of the PB2 or the PB2 and PB1 mutations, the three types of viral RNAs gradually reached the same levels as with the revertant virus (**Fig 3A–3C**).

The low vRNA to cRNA ratio observed for the att-PxW virus (**Fig 3D,** dotted bar compared to black bars for PR8) pointed to a defect in cRNA-> vRNA replication. Of note, in cells infected with the revertant virus the vRNA to cRNA ratio remained low compared to PR8 (**Fig 3D**, dark grey bar); however this is explained by the fact that vRNA levels increased back to PR8 levels (**Fig 3A**) whereas cRNA levels exceeded PR8 levels (**Fig 3B**). The fact that high levels of all three species of viral RNAs (vRNAs, cRNAs and mRNAs) are restored but with a lower than wild-type efficiency of the cRNA to vRNA synthesis step underlines the functional plasticity of the FluPol, and is consistent with the observation that the rev-PxW virus grows significantly better than the att-PxW virus but not quite as efficiently as the wild-type PR8 virus (**Fig 1B**). The mRNA to vRNA ratios observed for the PR8 and att-PxW virus showed no statistically significant difference (**Fig 3E**), suggesting that transcriptional activity of the att-PxW virus is unaffected. Nevertheless, when NP mRNA levels were measured under primary transcription conditions, using cycloheximide to inhibit *de novo* viral protein expression, they displayed an 8-fold reduction with the att-PxW reassortant compared to the wild-type PR8 (**Fig 3F**). In the presence of the single PB2-701 mutation they increased and reached the same levels as observed with the revertant, *i*.*e*. about 50% of the wild-type levels. Since the PB2-701 residue was shown to affect the nuclear import of PB2 and vRNPs [30, 31], and given our findings on mRNA levels and mRNA to vRNA ratios under transcription/replication conditions (**Fig 3C and 3E**), a defect in PB2/vRNP nuclear import rather than a defect in transcription most likely accounts for the reduced levels of primary mRNAs measured with the att-PxW virus. The PA-28 and PA-349 mutations, as well as the PB1-195 mutation, had no effect by their own and did not further increase primary transcripts levels when added to the PB2 mutation (**Fig 3F**).

Overall, the comparison of the viral growth kinetics of the PR8, att-PxW, rev-PxW viruses with those of the reverse-genetically rescued viruses bearing either single PB2, PB1 or PA mutations or combinations of the latter (**Fig 2B and 2C**) and measurements of the v-, c- and mRNA levels for the same viruses (**Fig 3**) pointed to the two second-site PA mutations being the most effective to compensate for the presence of the WSN-PB2 protein in an otherwise PR8 background, underlining the functional interplay between the PB2 and PA polymerase subunits.

### Mutations on PA compensate for a replication defect of the PR8xWSN-PB2 polymerase

**Fig 4:**
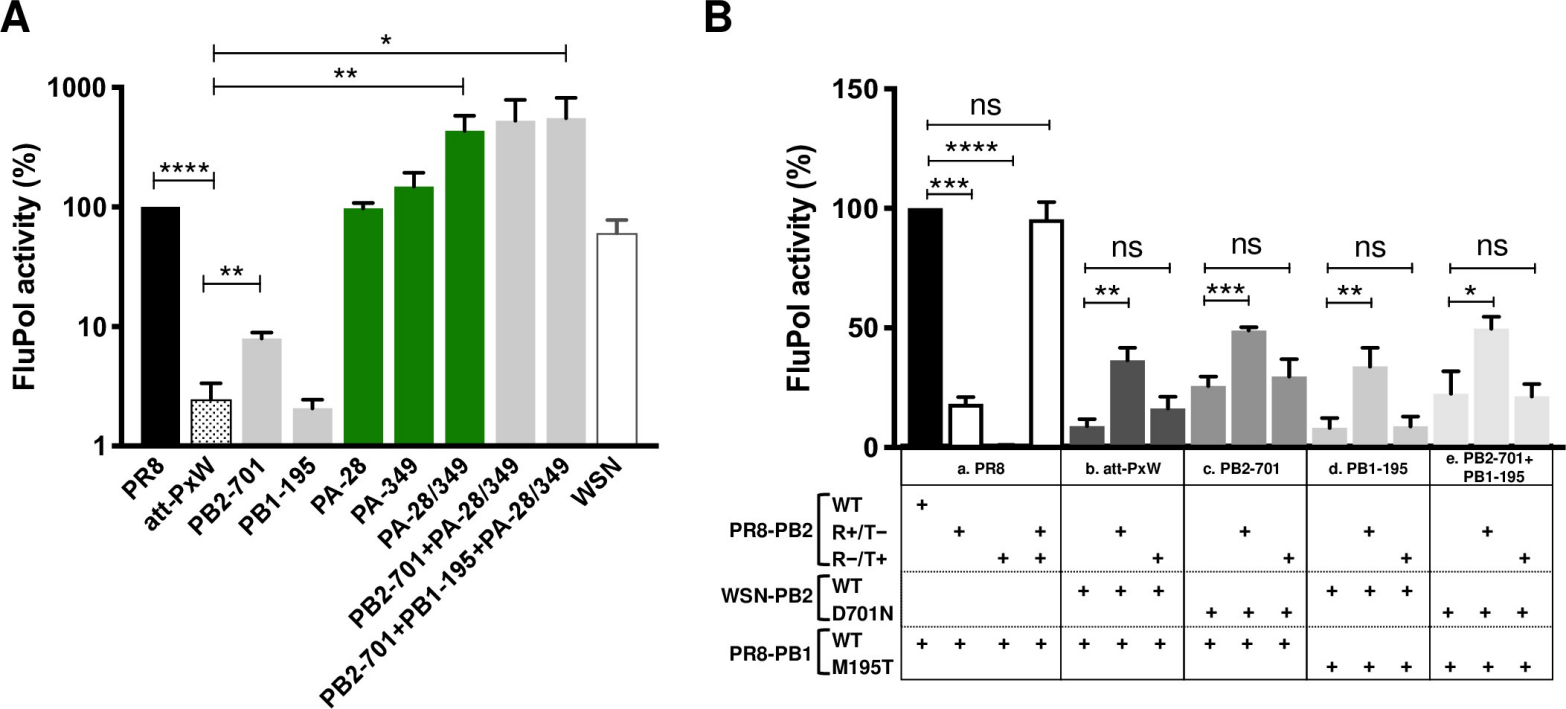
Polymerase activities measured in a minigenome assay. **(A)** Polymerase activities of mutant FluPols. HEK-293T cells were co-transfected with plasmids expressing the att-PxW polymerase, using wild-type (dotted bar) or mutant WSN-PB2 and/or PR8-PA plasmids (grey and green bars) as indicated, together with the PR8-NP, pPolI-Firefly and pTK-*Renilla* plasmids. Plasmids expressing the polymerase and NP from the PR8 (black bar) and WSN (white bar) viruses were used as controls. Firefly luciferase activities were measured at 24 hours post-transfection and normalized relative to *Renilla* luciferase activities. The results are expressed as percentages (PR8: 100%) and are shown as mean ± SD of three independent experiments in triplicates. Significance was assessed using one sample t-test (for pairwise comparison with the 100% PR8 reference) or Student’s unpaired t-test for pairwise comparison among other samples (*p ≤ 0.05; **p ≤ 0.01; *** p ≤ 0.001; **** p ≤ 0.0001). (**B**) Transcription/replication activity in the presence of *trans*-complementing transcription-deficient or replication-deficient PB2 proteins. HEK-293T cells were co-transfected with plasmids expressing the PR8 (**a**) or att-PxW (**b-e**) polymerase, together with the PR8-NP, pPolI-Firefly and pTK-*Renilla* plasmids. Where indicated, plasmids encoding a transcription-deficient (PB2-E361A, R+/T-) or replication-deficient (PB2-R142A, R-/T+) PB2 protein were co-transfected to *trans*-complement the att-PxW polymerase (**b-e**). As a control, they were transfected instead of the PR8-PB2 plasmid in (**a**). Firefly luciferase activities were measured at 48 hours post-transfection and normalized relative to *Renilla* luciferase activities. The results are expressed as percentages (PR8: 100%) and are shown as mean ± SD of three independent experiments in triplicates. Significance was assessed using a one sample t-test in (**a**) and Student’s unpaired t-test in (**b-e**) (**p ≤ 0.01; *** p ≤ 0.001; **** p ≤ 0.0001; ns: non significant).

To further investigate the molecular basis for PB2-mediated attenuation and PA-mediated reversion of the att-PxW reassortant, we assessed the activity of transiently reconstituted vRNPs in a minigenome assay. HEK-293T cells were co-transfected with plasmids allowing the expression of PB2, PB1, PA, and NP proteins, a pseudo-viral RNA containing the Firefly luciferase reporter gene, and the *Renilla* protein as an internal transfection control. The efficiency with which the viral-like RNA underwent transcription and replication was evaluated by the normalized Firefly/*Renilla* signal. Western blots were performed to verify that the wild-type and mutant FluPol subunits were expressed at similar levels upon transfection (**S1 Fig**). The wild-type PR8 and WSN vRNPs displayed similar activities (**Fig 4A**, black and white bars) whereas the att-PxW vRNPs reconstituted by the association of PR8-PB1, -PA and -NP together with WSN-PB2 showed a ≈ 40-fold lower activity (**Fig 4A**, dotted bar), in agreement with the attenuated phenotype of the att-PxW reassortant virus documented above (**Figs 1–3**). Each of the isolated PA-28 and PA-349 mutation, when introduced in the PxW background increased FluPol activity up to wild-type levels, which contrasted with their negative or moderately positive effect, respectively, on viral growth (**Fig 2B**). When combined, the PA mutations raised FluPol activity about 4-fold above wild-type (**Fig 4A**). Therefore the minigenome assay partially recapitulated the contribution of the PA mutations initially observed in the infectious context, and confirmed the PB2-PA functional interplay. The PB2 and PB1 mutations had a moderate or no effect by their own, and did not further increase FluPol activity levels when added to the double-PA mutations (**Fig 4A**). This is consistent with the observation that the corresponding viruses bearing single PB2-701 or PB1-195 mutations display viral growth properties similar to the att-PxW virus (**Fig 2C**).

To further assess whether the chimeric PxW FluPol is impaired for transcription and/or replication, we *trans*-complemented it with a replication-competent/transcription-deficient (E361A, R+/T-, [10]) or a replication-deficient/transcription-competent (R142A, R-/T+, [32]) PR8-PB2 protein in the minigenome assay as described above. As a control, we checked that the two mutant PR8-PB2 proteins were expressed as efficiently as the wild-type PB2 (**S1A Fig**) and were able to *trans*-complement each other (**Fig 4B, a)**. *Trans*-complementation was then performed in the att-PxW FluPol background, in the absence (**Fig 4B, b**) or presence of the PB2-701 and PB1-195 and mutations, either isolated (**Fig 4B, c-d)** or in combination (**Fig 4B, e**). *Trans*-complementation with the transcription-defective but replication-competent PB2 resulted in a moderate but significant and systematic increase in Firefly/*Renilla* signals, whereas no statistically significant increase was observed upon *trans*-complementation with the replication-defective and transcription-competent PB2 (**Fig 4B**, **b-e**). These observations suggest that the chimeric PxW FluPol has a major defect in its replication activity that can be partially *trans*-complemented by the R+/T- PB2 E361A. They are in agreement with the decreased vRNA to cRNA ratios observed in the infectious context (**Fig 3D**). Taken altogether our data indicate that the PA-28 and PA-349 mutations observed in the revertant virus compensate for a major replication defect of the PxW FluPol.

### Three independent reversion patterns all affect FluPol-FluPol contact regions

**Fig 5:**
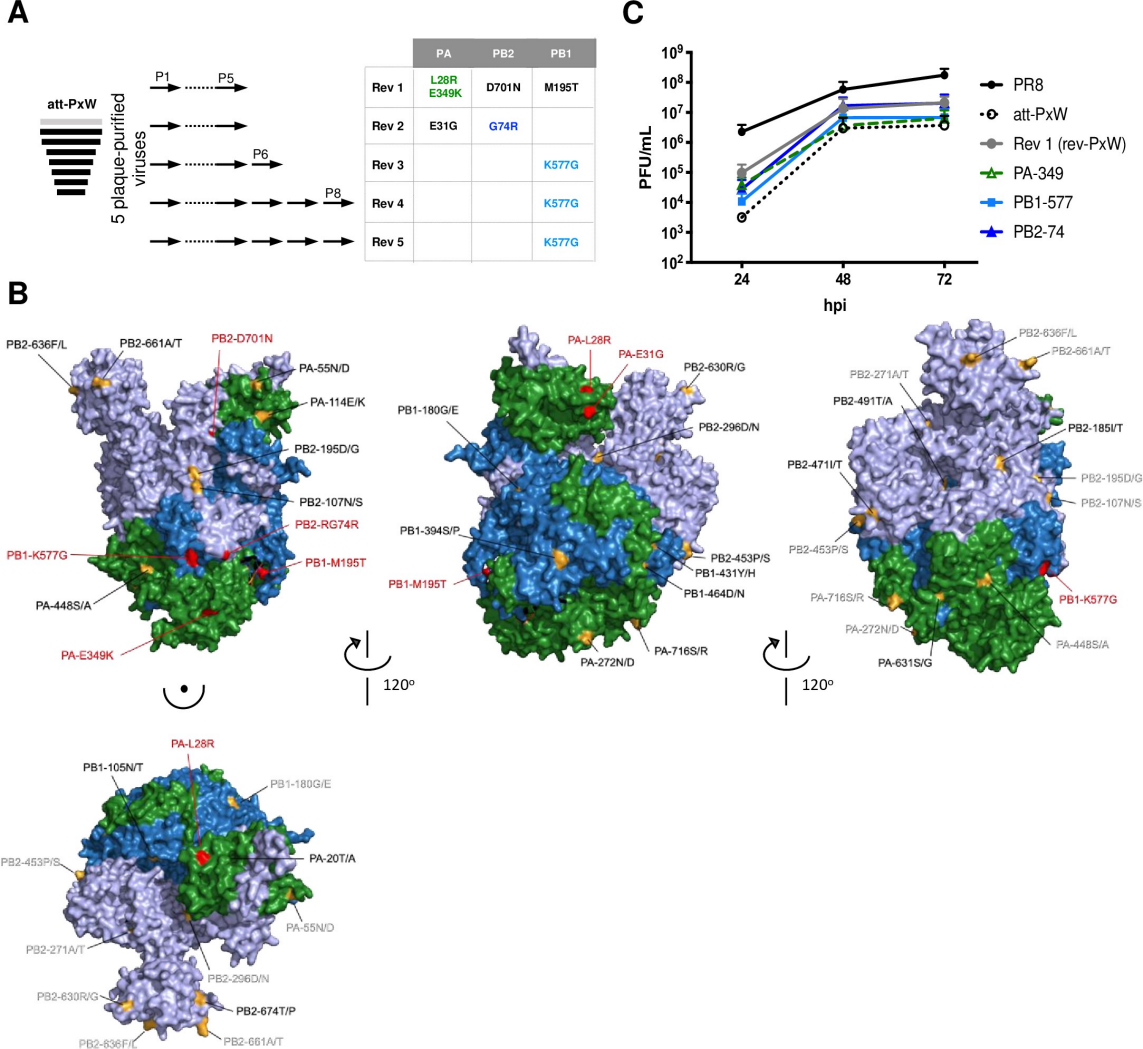
FluPol mutations observed on revertant viruses derived from five independent serial passages of the att-PxW virus. **(A)** Schematic representation of five independent serial passaging of plaque-purified att-PxW viruses. As soon as large plaque forming viruses were observed, at passages 5 to 8 (P5 to P8) as indicated, they were isolated by plaque purification and subjected to whole genome next-generation sequencing. Only reversion mutations on the FluPol genes that were present on >98% of the reads are indicated (other mutations are shown in **S2 Table**). **(B)** Mapping of amino-acids of interest onto the three-dimensional structure for a cRNA-bound FluPol (influenza B/Memphis/13/03 virus, PDB: 5EPI). The PB2, PB1 and PA subunits are represented in purple, blue and green, respectively. The amino-acids that differ between the PR8 and WSN viruses are colored in bright orange and labelled as PR8/WSN residues. The amino acids mutated in the revertant viruses isolated upon serial passaging of the att-PxW virus are colored in red. Grey labeling indicates residues that can be clearly visualised in one of the previous views of the cRNA-bound FluPol structure in the figure. **(C)** Growth kinetics under multi-cycle conditions. A549 cells were infected at a m.o.i. of 0.001 with the indicated viruses. At the indicated times post-infection, viral titers were determined by plaque assay on MDCK cells. The results are shown as the mean ± SD of three independent experiments, except for the 72 h time point of Rev 1 that was only measured twice.

Four additional, independent serial passages of the att-PxW virus were performed (**Fig 5A**, Rev2 to Rev5). Viruses forming large plaques were detected at passages 5 to 8 and were isolated by plaque purification. Upon sequencing, a combination of PB2-G74R and PA-E31G was found in one revertant virus, and the identical PB1-K577G mutation was found in the three others (mutations are referred to below and in the figures as PB2-74, PA-31 and PB1-577). Residues PB2-G74, PA-E31 and PB1-K577 are conserved between the parental PR8 and WSN viruses.

Since our data on the att-PxW virus pointed to a defect in cRNA -> vRNA synthesis (**Fig 3D**), we positioned the mutated residues on the three-dimensional structure of a cRNA-bound FluPol [33]. The PA-28, PA-349 and PB1-195 residues which underwent mutation in the rev-PxW virus all appear to be surface-exposed, as are the PB2-74, PA-31 and PB1-577 residues (**Fig 5B**, colored in red). Strikingly, PB2-74, PB1-577 and PA-349 are all three located within FluPol-FluPol contact regions in the crystal and cryo-EM structures of a dimer of RNA-free FluPol [9]. The FluPol dimerization interface described by Chang et *al*. [8] also encompasses residue PA-349. The RNA-free FluPol (or apo-form) has a structure similar to that of the cRNA-bound form [4, 5, 33].

Recombinant viruses with isolated PA-31, PB2-74 or PB1-577 mutations or combined PA-31 and PB2-74 mutations, in the att-PxW background, were produced. Their genotypes were confirmed by whole genome NGS sequencing and their growth kinetics (**Fig 5C**) and plaque phenotypes (**S2A Fig**) were assessed. Remarkably, all three viruses with mutations in the FluPol-FluPol dimerization interface grew faster than the att-PxW virus, as indicated by 5- to 10-fold higher infectious titers at 24 hpi, getting close to those observed with the rev-PxW virus (**Fig 5C**, green and blue lines). Similarly to the PA-349 mutation, none of the single PB2-74 nor PB1-577 mutations were sufficient on their own to restore a wild-type phenotype in terms of growth kinetics (**Fig 5C**) or plaque phenotype (**S2A Fig**). The virus with the single PA-31 mutation, located very close to PA-28 on the apo FluPol 3D structure (**Fig 5B**), grew significantly slower than the att-PxW virus (**S2B Fig**), as did the PA-28 mutant virus (**Fig 2B**). Notably however, while the virus bearing the PA-28 in addition to the PA-349 mutation displayed a significant growth advantage over the single PA-349 mutant virus (**Fig 2B**), the virus bearing the PA-31 in addition to the PB2-74 mutation was attenuated compared to the single PB2-74 mutant virus (**S2B Fig**). We speculate that second site mutations present in viral subpopulations (as shown in **S2 Table**) may have additive or synergic effects with the PB1-577 and PB2-74/PA-31 mutations.

### Reversion mutations modulate dimerization of the viral polymerase

Based on functional studies of purified positive-sense complementary ribonucleoproteins (cRNPs), it was proposed that oligomerization between a FluPol in *trans* and the cRNP-associated FluPol was required for cRNA -> vRNA synthesis [11]. These data, taken together with our observed defect in cRNA -> vRNA replication for the att-PxW virus, and the localization of mutations at the FluPol-FluPol dimerization interface in the revertants, prompted us to investigate the dimerization profile of the att-PxW FluPol.

**Fig 6:**
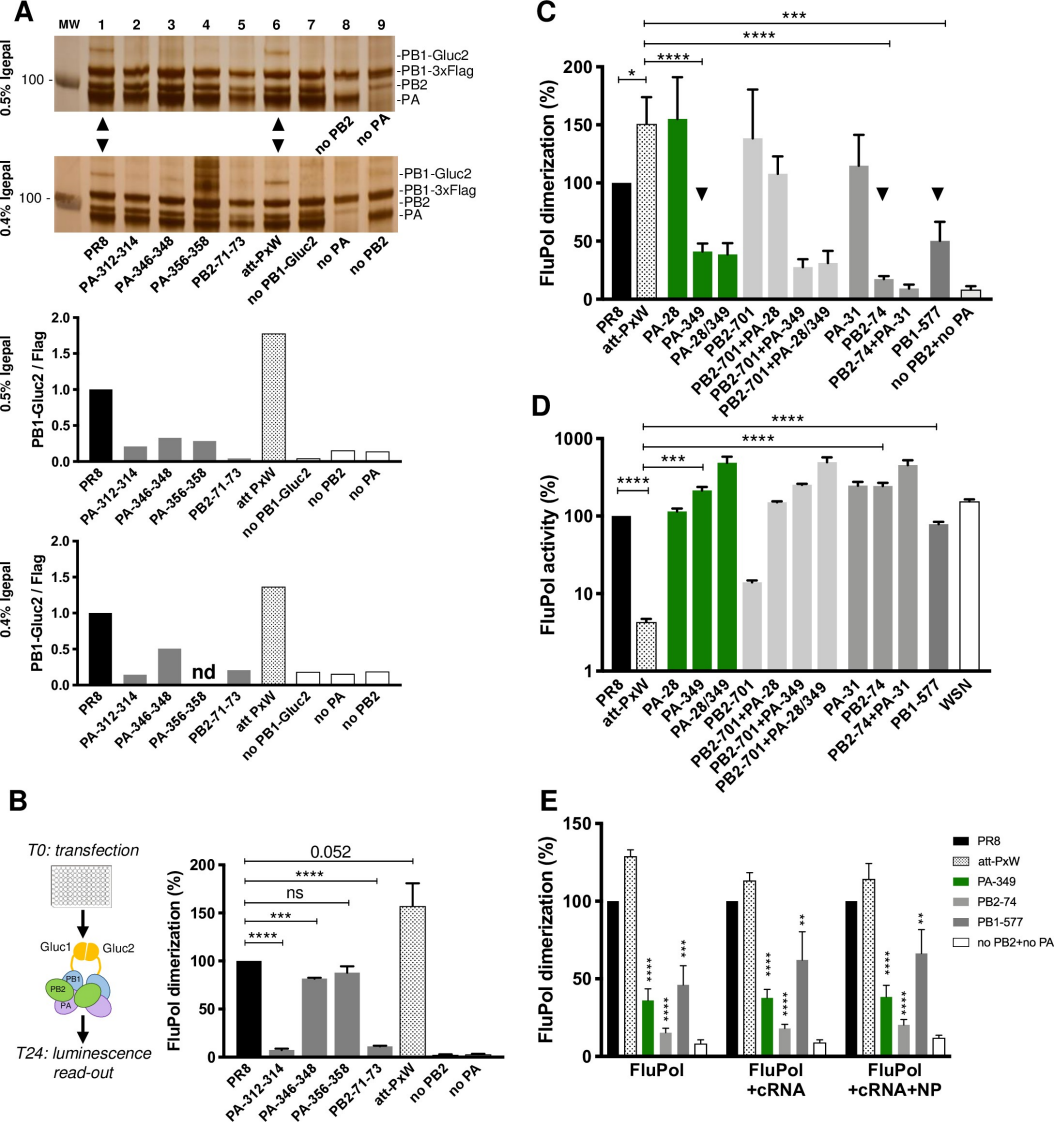
Dimerization of mutant FluPol heterotrimers. **(A)** FluPol dimerization assessed by co-immunoprecipitation. HEK-293T cells were co-transfected with plasmids encoding the PR8 polymerase (both PB1-3xFlag and PB1-Gluc2, together with the wild-type or mutant PR8-PA and PR8-PB2 as indicated, lanes 1-5), or the att-PxW polymerase (both PR8-PB1-3xFlag and PR8-PB1-Gluc2 together with PR8-PA and WSN-PB2, lane 6). Controls in the absence of PB1-Gluc2, PA or PB2 were also performed (lanes 7-9). FluPol complexes were purified at 48 hours post-transfection using anti-Flag antibody-magnetic beads, in the presence of either 0.5 (upper panel) or 0.4% (lower panel) Igepal CA-630 and analysed by SDS-PAGE and silver staining. Molecular weight markers are indicated. Quantification of the PB1-Gluc2 and PB1-3xFlag signals was performed by gel densitometry on a BioRad ChemiDoc Imaging apparatus, using the BioRad software. The PB1-Gluc2/PB1-3xFlag ratios are presented, with the wild-type PR8 condition used as a reference (PR8-PB1-Gluc2/PR8-PB1-3xFlag ratio: 1). MW: Molecular Weight Marker (kDa). **(B)** Schematic representation of the split luciferase complementation-based assay for FluPol dimerization and validation of the assay for FluPol dimerization. The split luciferase Gluc1 and Gluc2 domains only interact to reconstitute an active luciferase enzyme when two fully reconstituted FluPol heterotrimers are associated. HEK-293T cells were co-transfected with the same plasmid combinations as in (A) except for PR8-PB1-3xFlag which was replaced by PR8-PB1-Gluc1. After 24 hours, the *Gaussia princeps* luciferase activities were measured. **(C)** FluPol dimerization for the wild-type PR8 and att-PxW FluPols, as well as for att-PxW FluPols bearing one or several of the reversion mutations observed in revertant viruses Rev 1 (green and light grey bars), Rev2 (medium grey bar), and Rev3-4-5 (dark grey bar), assessed as in (B). **(D)** Polymerase activity of the same series of FluPols as in (C), plus the WSN FluPol (white bar) assessed using a minigenome assay as described previously in **Fig 4A**. **(E)** FluPol dimerization in the context of a cRNP. FluPol dimerization was measured for the Apo FluPol and compared to dimerization in the presence of NA-cRNA or NA-cRNA + NP, for the att-PxW, PA-349, PB2-74 and PB1-577 FluPol complexes. (**B-E**) The results are expressed as percentages (PR8: 100%) and are shown as mean ± SD of three (B, D and E) or four (C) independent experiments in triplicates. Significance was assessed using one sample t test (for pairwise comparison with the 100% PR8 reference) and Student’s unpaired t-test for pairwise comparison among other samples (*p ≤ 0.05; **p ≤ 0.01; *** p ≤ 0.001; **** p ≤ 0.0001; ns: non significant). In (E), the significance of pairwise comparison with att-PxW in each experimental condition is indicated.

To this end, we first used a FluPol dimerization assay based on co-immunoprecipitation (co-IP) as previously described by others [7, 34]. We transiently reconstituted heterotrimeric PR8 FluPol complexes in HEK-293T cells, in the presence of both PB1-3xFlag and PB1-Gluc2 tagged proteins. A series of triple-alanine mutants of PA and PB2, expected to disrupt the dimerization interface as present in the crystal structure [9] (PA residues 312–314, 346–348, 356–358 and PB2 residues 71–73), were compared to their wild-type counterparts. The wild-type and mutant FluPol subunits were expressed at similar levels upon transfection as shown by western blot (**S1 Fig**). Two different lysis buffers were used for the co-IPs, including either 0.5 or 0.4% Igepal CA-630 and allowing for comparison of different stringencies (**Fig 6A,** upper and lower panels, respectively). Silver staining analysis revealed that PB1-Gluc2 co-purified with PB1-3xFlag in the presence of the wild-type PB2 and PA proteins, but not if PB2 or PA was omitted (**Fig 6A**, lane 1 compared to lanes 8–9), which confirmed that the co-purification of both tagged PB1 proteins was an indicator for FluPol heterotrimer dimerization. Replacing wild-type PB2/PA with any of the mutant PB2/PA proteins strongly decreased the amount of co-purified PB1-Gluc2 (**Fig 6A**, lanes 2–5 compared to lane 1). Interestingly, replacing PR8-PB2 with WSN-PB2 (as in the att-PxW virus) did not affect the co-purification of PB1-Gluc2, indicating that the att-PxW FluPol is not impaired for dimerization (**Fig 6A**, lane 6 compared to lane 1, arrowheads). Of note, the co-IP performed in less stringent conditions, i.e. 0.4% Igepal, seemed to provide a more differentiating assessment of mutants PA-312-314, PA-346-348 and PB2-71-73 (**Fig 6A**, lanes 2, 3 and 5 compared in upper and lower panels, and corresponding densitometry data shown below). Unfortunately the PA-356-358 mutant repeatedly produced a strong background in the 0.4% Igepal condition (**Fig 6A**, lane 4 in the lower panel) which precluded accurate characterisation by densitometry scanning.

We then adapted the assay for use in microtiter plates, by replacing the tagged PB1-3xFlag by PB1-Gluc1 and using *Gaussia princeps* (Gluc) split-luciferase complementation as a read-out (Materials and Methods section and schematic in **Fig 6B**). The results obtained with the above-described triple-alanine dimerization-deficient mutants of PA and PB2 were consistent with those obtained in the co-IP assay, *i*.*e*. they showed a strong reduction of the dimerization signal in the presence of the PA-312-314 and PB2-71-73 dimerization mutants compared to the wild-type PR8 and att-PxW, and a lower reduction in the presence of the PA-346-348 mutant (**Fig 6B** compared to **Fig 6A,** lower gel panel and the corresponding densitometry data). The split-luciferase assay may rely on a very low stringency lysis buffer as compared to the co-IP one, which would account for the relatively high dimerization signals observed with the PA-346-348 and PA-356-358 mutants (**Fig 6B**) and suggests that the corresponding residues are less critical for PR8-FluPol dimerization than residues PA-312-314 and PB2-71-73.

Finally we checked whether a similar FluPol dimerization signal could be detected in an infectious context, by using recombinant WSN viruses that express fusion PB1-Gluc1/2 or PB2-Gluc1/2 proteins [35], in the presence or absence of a co-expressed nanobody that binds next to the dimerization interface (nanobody Nb8205 [9]–plasmid kindly provided by E. Fodor and J. Steyaert). As described previously [35], a very high luciferase activity was measured in HEK-293T co-infected with a pair of PB1/PB2 tagged viruses (**S3A Fig**, upper panel, white hatched bars). Luciferase activity could also be measured upon infection with both PB1 tagged viruses, at a 10-fold lower level compared to PB1/PB2 tagged viruses but still >1000-fold higher than background, therefore likely reflecting FluPol dimerisation (**S3A Fig**, upper panel, white open bar). Given the proposed role of FluPol dimerization in viral replication [10, 11], transfection of the nanobody Nb8205 prior to infection was expected to decrease the overall production of viral proteins: indeed a lower signal was detected upon western-blot analysis with an anti-Gluc antibody, and a two-fold decrease of the PB1-PB1 and PB1-PB2 signals was observed (**S3A Fig**, upper and lower panels, open and hatched grey bars).

Overall our data validate the split-luciferase transfection assay as a relevant, specific and quantitative assay for FluPol dimerization. Consequently, it was then used to assess the effect of reversion mutations on dimerization of the att-PxW FluPol. Strikingly, while the FluPol dimerization signal measured for att-PxW was on average increased 1.5-fold compared to the wild-type PR8 (**Fig 6B and 6C,** dotted bars), it was significantly decreased in the presence of the PA-349, PB2-74 or PB1-577 mutations, down to 40%, 20% and 50% of the PR8 signal, respectively (**Fig 6C,** arrowheads). The more pronounced reduction in FluPol-FluPol dimerization detected in the presence of the PB2-74 mutant (**Fig 6C**) was consistently observed in the co-IP setting (**S3B Fig**). Western blot controls for polymerase expression in the split-luciferase experiment confirmed that the differences observed in the assay can be attributed to dimerization as no major difference in the steady-state accumulation of wild-type and mutant FluPol subunits was observed (**S3C Fig**).

In the minigenome assay performed in parallel, the PA-349, PB2-74 and PB1-577 mutations all restored high FluPol activity (**Fig 6D**). The other mutations (PB2-701, PA-28, PA-31), which are located outside the FluPol dimerization interface in the crystal and cryo-EM structures [9] did not affect the FluPol dimerization signal whether tested alone or in combination with the PA-349 or PB2-74 mutation, as shown in **Fig 6C**. In contrast, in the minigenome assay (**Fig 6D**), the PA-E31G mutations increased FluPol activity, as observed previously with the PA-28 (**Fig 4A**), to the same extent as did the PA-349, PB2-74 or PB1-577 mutations. An additive effect of PA-31 + PB2-74 was also observed, similar to that seen for PA-28/349 (**Fig 6D**). Since the FluPol-FluPol interaction is related to the cRNA -> vRNA synthesis step, we performed the split-luciferase dimerization assay in the presence of a viral cRNA, *i*.*e*. by co-transfecting a reverse genetics pPolI-WSN-NA-cRNA plasmid, or in the presence of a viral cRNA and co-expressed NP (**Fig 6E**). In order to mimic the binding of an Apo FluPol to a cRNP-associated FluPol, we inserted promoter binding mutations (M356A-E358A) in the PB1-Gluc2 construct. In addition, we inserted PB1 active site mutations (D445A-D446A) in both Gluc1- and Gluc2-tagged PB1 proteins, to avoid interference by FluPol activity in the FluPol dimerization read-out. We verified that each of the PB1-356/358 and PB1-445/446 mutants reduced FluPol activity to background levels in the minigenome assay (**S3D Fig**). Whether measured in the absence or presence of cRNA or cRNA + NP, the differences in FluPol dimerization between the wild-type and mutant PA-349, PB2-74 and PB1-577 FluPols were very similar (**Fig 6E** and **S3E Fig**). Therefore, the inverse correlation observed between FluPol dimerization and FluPol activity (**Fig 6C** compared to **Fig 6D**) is not merely due to the presence of viral RNAs and NP in the minigenome assay.

Our data demonstrate that second-site mutations on PA, PB1 or PB2 which contribute to the phenotypic reversion of the att-PxW virus inversely modulate the FluPol activity and FluPol dimerization. The significance of these findings with regards to function and regulation of the different conformational states of FluPol is discussed below.

## Discussion

The recently elucidated high-resolution structures for RNA polymerases of influenza A,B, C and D viruses have considerably enhanced our mechanistic understanding of viral transcription and genome replication [3–7, 33]. However, many aspects of FluPol function remain to be elucidated. For instance, what the molecular bases are for compatibility/incompatibility between non-cognate FluPol subunits upon genetic reassortment, or how interactions between heterotrimeric polymerases function to promote efficient replication remain unclear. Cryo-EM structures are available for dimers of a partial FluPol [8] and a whole FluPol [9] of influenza A viruses. A recently reported novel binding site for the 3’end of viral template RNAs lies close to the dimerization interface [4, 9], and a mechanistic model for a regulatory role of FluPol dimerization was recently proposed [9]. However so far there is limited evidence in an infectious context that the available dimeric structures represent a functional oligomerization state.

We found that a recombinant PR8xWSN-PB2 reassortant virus displayed a strongly attenuated phenotype, despite a 97% homology between the PR8-PB2 and WSN-PB2 proteins. To understand why, and rather than systematically mutating the residues that differ between the PR8 and WSN strains and thereby likely affecting the polymerase function at multiple steps of transcription, replication and/or progeny RNP assembly, we characterized natural revertant viruses selected upon serial passages. We observed three reversion patterns corresponding to distinct sets of second-site mutations. Remarkably, all revertant viruses have in common that one of the second site mutation, at residue PA-349, PB2-74 or PB1-577, is located within the FluPol dimer interface as observed in [9]. The residue PA-349 also appears to be within a FluPol dimer interface as described by Chang *et al*. [8]. None of the residues PA-349, PB2-74 or PB1-577 differ between the PR8 and WSN viruses. Remarkably however, in the PR8xWSN-PB2 genetic background, each of the PA-E349K, PB2-G74R and PB1-K577G mutations affects FluPol dimerization and restore wild-type-like FluPol activity in a minigenome assay. By further documenting the PA-349 mutation in the infectious context, we showed that it enhances the synthesis of viral RNAs, which is impaired for the PR8xWSN-PB2 reassortant, and enhances the production of infectious viral particles. Therefore, our data provide new strong genetic evidence for the functional importance of FluPol dimerization, as well as functional validation of residues located at the FluPol-FluPol interface in the dimer crystal and cryo-EM structures from Fan *et al*. [9]. They demonstrate that this particular dimeric conformation of FluPol is involved in viral RNA replication.

Moreover, and most importantly, our data reveal that a fine-tuned level of FluPol dimerization is required for optimal IAV replication. Although residues at the FluPol interface do not differ between the PR8 and WSN parental strains, the level of FluPol dimerization observed for the att-PxW virus was slightly (1.5-fold) higher than for the wild-type PR8 virus as measured in a cell-based assay. Strikingly, reversion mutations at residues PA-349, PB2-74 and PB1-577 all three reduced dimerization 2- to 5-fold below wild-type. A likely interpretation is that to ensure efficient replication, a correct balance between FluPol dimers and alternative FluPol conformation(s) is needed. We hypothesize that the att-PxW virus is deficient for one or some of these alternative conformations and that mutations that decrease the att-PxW FluPol dimerization levels rectify the conformational imbalance, allowing to reach optimal production of c-, v- and m-RNAs. According to this model, true reversion mutations in WSN-PB2, that would allow to readjust the optimal balance of alternative conformation(s), could also have been selected upon serial passages. Twenty-four residues differ between the PR8-PB2 and WSN-PB2 proteins, mostly located at hinge regions in between subdomains (**S4 Fig** and **Fig 5B**). Therefore, a complex pattern of reversion mutations on WSN-PB2 is possibly needed to recover a PR8-PB2-like function. In our study, five out of five independent reversion events corresponded to mutations within the FluPol interface. Although the number of independent events is too low to reach a final conclusion, our observations suggest that, in the att-PxW genetic background, modulation of the FluPol interface represents the most direct evolutionary pathway towards enhanced FluPol activity.

Two types of reversion patterns were observed in our study: either a single mutation within the FluPol dimer interface, at residue PB1-577, or a mutation within the FluPol dimer interface, at residue PA-349 or PB2-74, combined with a mutation in the N-terminal domain of PA at residue PA-28 or PA-31. The first pattern, *i*.*e*. the PB1-577 mutation, was observed in three out of five independent reversion events; it reduces FluPol dimerization levels to a lesser extent compared to the PA-349 and PB2-74 mutations (50%, compared to a 60% and 80% reduction, respectively) while restoring wild-type FluPol activity. We speculate that (i) unlike the PB1-577 mutation, the PA-349 and PB2-74 mutations might result in a sub-optimal level of dimerization and/or might impair another aspect of the FluPol function, and (ii) the mutations at residues PA-28 or PA-31 might have been selected to compensate for these drawbacks. Indeed, in the infectious context we found that the PA-28 mutation has an additive effect with the PA-349 mutation but is not beneficial on its own, although in the simplistic minigenome assay the PA-28 mutations enhances FluPol activity, as does the PA-31 mutation. Of note, discrepancies between FluPol activity as measured in a minigenome assay and viral growth capacity have been documented by others [22, 23]. The PA-28 and PA-31 residues are exposed at the surface of the PA endonuclease domain [36], irrespective of whether vRNA, cRNA or no RNA is bound to the FluPol. They lie very close to each other, and the PA-E31G and PA-L28R mutations alter the surface charge distribution in the same direction, from negative/neutral to neutral/positive. We speculate that the isolated PA-28 and PA-31 mutations may negatively affect FluPol functional interaction with a viral protein that is not expressed in the minigenome assay (e.g. NS1 or NEP), or the function of the PA-X protein that is known to modulate the host immune responses [37]. In the particular context of the revertant viruse harbouring the PA-349 mutation, the negative impact of the PA-28 mutation could be either reduced or compensated by a stronger positive impact otherwise.

Our findings show that the polymerase subunits of a given viral strain or lineage co-evolve to ensure not only optimal inter-subunit interactions within the heterotrimer but also proper levels of dimerization of the heterotrimer. Co-segregation patterns that have been observed among the FluPol genes as an outcome of genetic reassortment [21, 23, 25] might also, at least in some cases, be related to keeping an optimal balance between FluPol monomers and oligomers. Detailed mechanistic studies would be required, in each case, to assess such hypothesis. In this regard, we show that the WSN-PB2 protein is incompatible with the PR8-PB1 and PR8-PA proteins. Interestingly, we found that the PR8xWSN-PB2 and WSNxPR8-PB2 situations are not symmetrical, *i*.*e*. the PR8-PB2 protein remains functionally compatible with WSN-PB1 and WSN-PA (**S5 Fig**), pointing to a complex interplay of genetic determinants. Non-reciprocal incompatibilities among FluPol subunits have been observed in other viral backgrounds [21, 38].

Finally, our study highlights the potential of mutations at the FluPol dimer interface in correcting a functional imbalance due to reassortment among the polymerase segments, despite the fact that residues located within the dimer interface are highly conserved. Among a set of > 3000 seasonal human IAVs, deviation from the consensus sequence at residues PB1-577, PA-349, and PB2-74 (**S1 Table**) was found for only a few viruses (3, 9 and 1 viruses, respectively). Further studies are required to assess the functional significance of each of these sequence polymorphisms in its particular genetic context, and to further document how interactions between heterotrimeric polymerases function to promote efficient replication. Intriguingly, the PA-E349G and PB1-K577E mutations were found to contribute to enhanced polymerase activity and increased virulence of IAV in mice [39, 40], suggesting that the optimal FluPol conformational balance is not only determined by the FluPol gene constellation but also by the host-cell environment.

## Materials and methods

### Cells

A549 (provided by Pr. M. Schwemmle, Freiburg, Germany) and HEK-293T cells (provided by Dr. M. Perricaudet, Paris, France) were grown in complete Dulbecco's modified Eagle's medium (DMEM, Gibco) supplemented with 10% fetal bovine serum (FBS) and 1% penicillin-streptomycin. MDCK cells (provided by the National Influenza Center, Paris, France) were grown in Modified Eagle's medium (MEM, Gibco) supplemented with 5% FBS and 1% penicillin-streptomycin.

### Plasmids

The eight reverse genetics bidirectional plasmids for the rescue of the A/PR/8/34 (PR8) virus [29] were kindly provided by Dr. R. Fouchier (Erasmus MC, Rotterdam, The Netherlands). The unidirectional reverse genetics plasmids derived from the A/WSN/33 (WSN) virus [28] were a gift from Pr. G. Brownlee (Sir William Dunn School of Pathology, Oxford, UK). The viral WSN- and PR8-derived PB1, PB2, PA and NP open reading frames were amplified, using the reverse genetics plasmids as a template, and sub-cloned into the pCI plasmid (Promega). Site-directed mutagenesis to introduce mutations into the reverse genetics or expression plasmids was performed using the QuickChange II Site-directed Mutagenesis kit (Agilent Technologies) or the Q5 Site-Directed Mutagenesis Kit (New England Biolabs). Primers used for mutagenesis are available upon request. A pPolI-Firefly plasmid encoding the Firefly luciferase coding sequence in negative polarity flanked by the 5’ and 3’ non-coding regions of the NS segment (analogous to the pPolI-CAT plasmid described in [41]) and a pTK-*Renilla* plasmid were used for minigenome assays. The pPolI plasmid encoding the NA cRNA was constructed by amplifying the viral NA segment from the reverse genetics pPolI-WSN-NA plasmid [28] and subcloning it in the appropriate orientation in the pPR7 plasmid which contains a PolI promoter and the hepatitis delta ribozyme, as described in [42]. The split-Gaussia Luciferase pCI-PR8-PB1-LL-Gluc1 and pCI-PR8-PB1-LL-Gluc2 plasmids used for FluPol dimerization assays were constructed according to [35, 43]. The pcDNA 3.1 plasmid encoding the nanobody Nb8205 was kindly provided by E. Fodor (University of Oxford) and J. Steyaert (VIB, Flanders, Belgium). All constructs were verified by sequencing.

### Production and characterization of recombinant viruses

Recombinant influenza viruses were produced by reverse genetics as described previously [35]. The efficiency of reverse genetics was evaluated by titrating the supernatant on MDCK cells, in a standard plaque assay adapted from Matrosovich *et al*. [44]. Viral amplifications and serial passages were performed by infecting MDCK cells at a m.o.i. of 0.001 and incubating them for 3 days at 37°C in DMEM containing TPCK-Trypsin (Sigma) at a final concentration of 1 μg/mL. Plaque purification under agarose overlay was followed by a single round of amplification on MDCK cells. For next-generation sequencing of the full viral genome, viral RNA was extracted from 140 μL of viral stocks using the QIAamp Viral RNA Mini kit (Qiagen) and eluted in 50 μL of nuclease-free water. Reverse transcription and amplification of the eight genomic segments were performed using the RT-PCR protocol adapted by the National Influenza Center (Institut Pasteur) from Watson *et al*. [45]. Briefly, 5 μL of viral RNA were subjected to RT-PCR using the Superscript One-Step RT-PCR with Platinium Taq kit (Thermo Fisher) and primers specific to the extremities conserved in all segments (U12/U12G and U13, described in **S3 Table**). PCR products were purified using the Nucleospin PCR Clean-up Kit (Macherey Nagel) and quantified using the Quant-iT PicoGreen dsDNA Assay Kit (Thermo Fisher). Next-generation sequencing was performed by the P2M facility at Institut Pasteur, using the Nextera XT DNA Library Preparation kit (Illumina), the NextSeq 500 sequencing systems (Illumina), and the CLC Genomics Workbench 9 software (Qiagen) for analysis.

### Quantification of v-, c-, mRNAs by strand-specific RT-qPCR

For single-cycle growth assays, A549 cells were seeded in 24-well plates (2.5x10^5^ cells) one day prior to infection at a m.o.i of 5, in the absence or presence of 100 μg/mL cycloheximide (Sigma). At the indicated time-points, cells were rinsed and total RNA was extracted using the RNeasy mini Kit (Qiagen). For the quantification of viral v-, c- and mRNAs, we adapted the strand-specific RT-qPCR protocol described in [46], using the primers listed in **S3 Table**. Briefly, cDNAs were synthesized with strand-specific RT primers tagged at their 5′ end with the hot-start modification of using saturated trehalose: a 5.5 μL mixture containing 200 ng of total RNA sample and 10 pmol of tagged RT primer was heated for 10 min at 65°C, chilled immediately on ice for 5 min, and then heated again at 60°C. After 5 min, 14.5 μL of a preheated reaction mix (4 μl First Strand buffer 5X (Thermo Fisher), 1 μL 0.1 M dithiothreitol (DTT), 1μL dNTP mix (10 mM each), 1μL Superscript III reverse transcriptase (200 U/μl, Thermo Fisher), 1 μL RNasin Plus RNase inhibitor (40 U/μL, Promega) and 6.5 μL saturated trehalose) was added and incubated at 60°C for 1 h.

Real-time PCR (qPCR) was performed with the Brilliant II SYBR Green qPCR Master Mix (Agilent Technologies) on a LightCycler 480 (Roche). Seven microliters of a 10-fold dilution of the cDNA was added to the qPCR reaction mixture (10 μL Brilliant II SYBR Green qPCR Master Mix 2X, 1.5 μL of each qPCR primer at 10 μM). The cycle conditions of qPCR were 95°C for 5 min, followed by 50 cycles of 95°C for 15 s, 56°C for 15 s and 60°C for 45 s. Synthetic v-, c- and mRNAs for the NP segment were synthesized by *in vitro* transcription with the MEGAscript T7 Transcription Kit (Thermo Fisher) and ten-fold serial dilutions (10^9^ to 10^3^ copies/μL) were used to generate standard curves.

### Multicycle growth assay

For multicycle growth assay, A549 cells were seeded in 96-well plates (4 x 10^4^ cells/well) one day prior to infection at a m.o.i of 0.001. After 1 h adsorption at 37°C, Opti-MEM containing TPCK-Trypsin at a final concentration of 0.5 μg/mL was added. The supernatants were collected at 24, 48 and 72 hpi and were used for titration on MDCK cells as indicated above.

### Minigenome assays

HEK-293T cells were seeded in 96-well white opaque plates (Greiner Bio-One, 3x10^4^ cells/well) one day before being transfected with 25 ng of each of the pCI plasmids expressing the PB2, PB1, PA viral proteins, together with 50, 10 and 5 ng of the pCI-NP, pPolI-Firefly and pTK-*Renilla* plasmids, respectively, using the FuGENE-HD transfection agent (Promega). The luciferase activities were measured at 24 hours post transfection using the Dual-Glo Luciferase Assay system (Promega) and a Centro XS LB960 microplate luminometer (Berthold Technologies). When minigenome assays were performed with the *trans*-complementing pCI plasmids expressing the PR8-PB2 mutants E361A and R142A, luciferase activities were measured at 48 h post-transfection.

### Immunoprecipitation-based polymerase dimerization assay

A protocol adapted from Nilsson-Payant *et al*. [34] was used to co-immunoprecipitate FluPol dimers. Briefly, 6 x 10^6^ HEK-293T cells seeded in a 10 cm culture dish were transfected one day after with 2.5 μg of each of the pCI-PB1-3xFlag and pCI-PB1-Gluc2 plasmids, together with 5 μg of each of the pCI-PB2 and pCI-PA plasmids, using polyethyleneimine (PEI, Polysciences Inc). After 48 h, cell lysates were prepared with 500 μL of lysis buffer (50 mM Tris-HCl pH 7.6, 200 mM NaCl, 25% glycerol, 0.5 or 0.4% Igepal CA-630, 1 mM DTT, 1X EDTA-free protease inhibitor cocktail tablet (Roche)) at 4°C for 30 min. After centrifugation at 16,000 g for 15 min to remove cell debris, cell lysates were incubated at 4°C for 6 h with 20 μL of anti-Flag M2 magnetic beads (Sigma), in a 1 mL volume adjusted with washing buffer (10 mM Tris-HCl pH 7.6, 150 mM NaCl, 10% glycerol, 0.1% Igepal CA-630, 1X EDTA-free protease inhibitor). The magnetic beads were then washed 6 times with washing buffer and elution was performed with 30 μL of 1X Laemmli buffer (Invitrogen). Purified proteins were analyzed by SDS-PAGE and revealed by silver staining (Sigma).

### Split luciferase complementation-based polymerase dimerization assay

HEK-293T cells were seeded in 96-well white opaque plates as described above, one day before being transfected with 10 ng of each the pCI-PR8-PB1-LL-Gluc1 and pCI-PR8-PB1-LL-Gluc2 plasmids together with 20 ng of each of the pCI plasmids expressing the PB2 and PA viral proteins, using PEI. To determine background signal, the PA or PB2 plasmid was omitted and the total amount of plasmid was adjusted to 60 ng using the empty pCI plasmid. After 24 h of incubation at 37°C, the luciferase enzymatic activity was measured using the *Renilla* Luciferase Assay system (Promega) and a Centro XS LB960 microplate luminometer. To perform the split-luciferase complementation assay in the cRNP context, 10 ng of each the pCI-PR8-PB1-445/446-LL-Gluc1-mutant and pCI-PR8-PB1-445/446-356/358-LL-Gluc2-mutants were co-transfected with 20 ng of each of the pCI-PB2 (PR8 or WSN, wild-type or mutant) and pCI-PR8-PA (wild-type or mutant) plasmids, in the absence or presence of 40 ng of the pCI-PR8-NP plasmid and in the absence or presence of 10 ng pPolI-WSN-NA-cRNA expressing plasmid, using PEI. Total amount of plasmid was adjusted to 110 ng with pcDNA.

### Human influenza polymerase protein sequence alignments

Amino acid sequences of human IAV PB2, PB1, and PA proteins were downloaded from the NCBI influenza database (https://www.ncbi.nlm.nih.gov/genomes/FLU/Database/nph-select.cgi?go=database). The following criteria were chosen: viral type: A; host: human; country/region: Northern temperate; sub type: any; full length only. Identical sequences were collapsed to limit sampling biases. Sequences with internal undetermined regions (represented as stretches of more than five X) were removed manually. After filtering, the total number of sequences was 4394, 3782 and 4391 for PB2, PB1 and PA, respectively. Protein sequences were aligned using the MAFFT multiple alignment tool (https://mafft.cbrc.jp/alignment/server/) and the amino acid polymorphism at each position was analysed using the Influenza Research Database “Analyze Sequence Variation” tool (https://www.fludb.org/brc/snpAnalysis.spg?method=ShowCleanInputPage&decorator=influenza).

## Supporting information

S1 Fig Western blot detection of transiently expressed wild-type and mutant FluPol proteins. HEK-293T cells were transfected with 50 ng of plasmids expressing the indicated wild-type of mutant FluPol proteins. Total cell lysates were prepared 24 h post-transfection in Laemli buffer and analysed by western blot as described in Diot et al. (Sci Rep 2016, doi: 10.1038/srep33763), using antibodies directed against PB2 (#GTX125925, GeneTex) **(A)**, PB1 (#PA5-34914, Thermo Fisher) **(B)** and PA (a gift from B. Delmas, INRA Jouy-en-Josas) **(C)**, or a GAPDH loading control antibody (#MA5-15738, Thermo Fisher). Molecular weight markers are indicated. Cropped blots are shown.
(PDF)

S2 Fig Characterization of recombinant viruses bearing reversion mutations. **(A)** Titers and plaque phenotypes of recombinant viruses bearing one or several reversion mutations in the att-PxW genetic background. The PB2-G74R, PA-E31G, PB2-G74R+PA-E31G, and PB1-K577G mutant viruses were rescued in parallel with the recombinant PR8 and att-PxW viruses. Following one round of amplification on MDCK cells, the titers and plaque phenotypes were compared to that of the rev-PxW virus. **(B)** Growth kinetics under multi-cycle conditions. A549 cells were infected at a m.o.i. of 0.001 with the indicated viruses. At the indicated times post-infection, viral titers were determined by plaque assay on MDCK cells. The results are shown as the mean ± SD of three independent experiments except for the 72 h time point of Rev 1 that was only measured twice.
(PDF)

S3 Fig FluPol dimerization assays. (**A**) Split luciferase complementation-based assay in an infectious context. Upper panel: HEK-293T cells were transfected with 80 ng of pcDNA3.1 plasmid encoding the nanobody Nb8205 (grey bars) or empty pcDNA 3.1 (white bars) as a control and 24 hours later they were infected at a m.o.i. of 5 with the indicated combinations of recombinant WSN viruses expressing fusion PB1-Gluc1/2 and/or PB2-Gluc1/2 proteins [35] to assess either FluPol dimerization or heterotrimer formation through the PB1-PB1 (open bars) or PB1-PB2 (hatched bars) interactions, respectively. After 6 h of incubation at 37°C, the luciferase enzymatic activity was measured. Western blots to verify expression of the Gluc-tagged PB1/PB2 proteins or 6xHis-tagged nanobody were performed using antibodies directed against Gluc (#E8023S, New England Biolabs) and His-tag (NPB1-41288, Novus Biologicals). The results of one representative experiment (mean of technical triplicates) are shown. Lower panel: the luciferase signals for the PB1-PB1 interaction representative of FluPol dimerization (open bars, mean ± SD of three independent experiments) and for the PB1-PB2 interaction (hatched bars, mean ± SD of two independent experiments), are represented as ratios of signal in the presence of the nanobody Nb8205 (grey bars) over empty pcDNA 3.1 control (white bars). (**B**) FluPol dimerization assessed by co-immunoprecipitation. HEK-293T cells were co-transfected with plasmids encoding the PR8 polymerase (both PB1-3xFlag and PB1-Gluc2, together with the wild-type PR8-PA and PR8-PB2), the att-PxW polymerase (both PR8-PB1-3xFlag and PR8-PB1-Gluc2 together with PR8-PA and WSN-PB2) or different combinations of the FluPol bearing the reversion mutations in the att-PxW background, as indicated. In the case of the PB1-577 mutation, the K577G mutation was introduced into the PR8-PB1-Gluc2 and PR8-PB1-3xFlag expression plasmids. Controls in the absence of PA or PB2 were also performed. FluPol complexes were purified at 48 h post-transfection using anti-Flag antibody-magnetic beads, in the presence of 0.4% Igepal CA-630 and analyzed by SDS-PAGE and silver staining. MW: Molecular Weight marker (kDa). Dashed lines separate distinct parts of the same gel. (**C**) Western blot detection of the wild-type and mutant FluPol proteins expressed in the split-luciferase complementation assay of **Fig 6C** using antibodies directed against Gluc, PB2 (#GTX125925, GeneTex) and PA (a gift from B. Delmas, INRA Jouy-en-Josas) or a GAPDH loading control antibody. Cropped blots are shown. (**D**) Polymerase activities of mutant FluPols. HEK-293T cells were co-transfected with plasmids expressing the wild-type PR8 FluPol heterotrimer or FluPol heterotrimers with either PB1 active site mutations (D445A/D446A, noted PB1-445/446) or PB1 template binding mutations (M356A/E358A or H32A/T34A, noted: PB1-356/358 and PB1-32/34 respectively) as indicated, together with the PR8-NP, pPolI-Firefly and pTK-*Renilla* plasmids. Firefly luciferase activities were measured at 24 h post-transfection and normalized relative to *Renilla* luciferase activities. The results of one experiment (mean of technical triplicates, expressed as percentages, PR8: 100%) are shown. Western blot detection of the wild-type and mutant PB1 proteins expressed in the minireplicon assay using an antibody directed against PB1 (#PA5-34914, Thermo Fisher) or of GAPDH are shown in cropped blots. Of note, only the PB1-356/358 template binding mutant was used in the split-luciferase complementation assay shown in **Fig 6E**. NC: Negative Control (no PB1 plasmid). (**E**) Western blot detection of NP protein as well as the wild-type and mutant Gluc-tagged PB1 proteins expressed in the split-luciferase complementation assay shown in **Fig 6E** was performed using antibodies against Gluc and NP (polyclonal antibodies against influenza A\PR\8\34 virus), respectively. Western blots against GAPDH as a loading control were also performed for each blot. Cropped blots are shown.
(PDF)

S4 Fig Mapping of the amino acid differences between PR8 and WSN FluPol subunits. Residues that differ between the PR8 and WSN viral polymerase subunits were mapped on linear representations of the PB2, PB1 and PA protein subdomains (adapted from [4]). The residues that undergo conservative, semi-conservative and non-conservative changes are indicated (amino acid found in PR8/WSN) in black, blue and red, respectively.
(PDF)

S5 Fig The WSNxPR8-PB2 reassortant virus is not attenuated. **(A)** Titers and plaque phenotype of wild-type PR8 and WSN, and reassortant PR8xWSN-PB2 (PxW-PB2) and WSNxPR8-PB2 (WxP-PB2) viruses. The viruses were rescued by reverse genetics and their titers and plaque phenotypes were determined as described in **Fig 1**. **(B-D)** Levels of NP v-, c- and mRNAs determined at 6 hpi by strand specific RT-qPCR in A549 cells infected at a m.o.i. of 5. The copy numbers determined as described in **Fig 3** are shown as the mean ± SD of three independent experiments in duplicate. **(E)** Accumulation of primary NP transcripts in the presence of cycloheximide. The mRNA/vRNA ratios are the mean ± SD of 3 independent experiments in duplicates. **(F)** Activity of the indicated vRNPs as measured in a minigenome assay. The transfections and luciferase read-outs were performed as described in **Fig 4A**. The results are expressed as percentages (PR8: 100%) and shown as means ± SD of three independent experiments in triplicates.
(PDF)

S1 Table Analysis of sequence variation at each mutation site among a subset of human seasonal IAVs. (PDF)

S2 Table Mutations detected upon whole genome sequencing of revertant viruses by NGS, present in >10% of total reads, at a given position. (PDF)

S3 Table Primers used in this study. (PDF)
