# Supplementary figures and images for: Influenza virus polymerase subunits co-evolve to ensure proper levels of dimerization of the heterotrimer
Source: PLoS Pathog. 2019 Oct 3;15(10):e1008034. doi: 10.1371/journal.ppat.1008034 (PMC6776259; doi:10.1371/journal.ppat.1008034)

S1 Fig

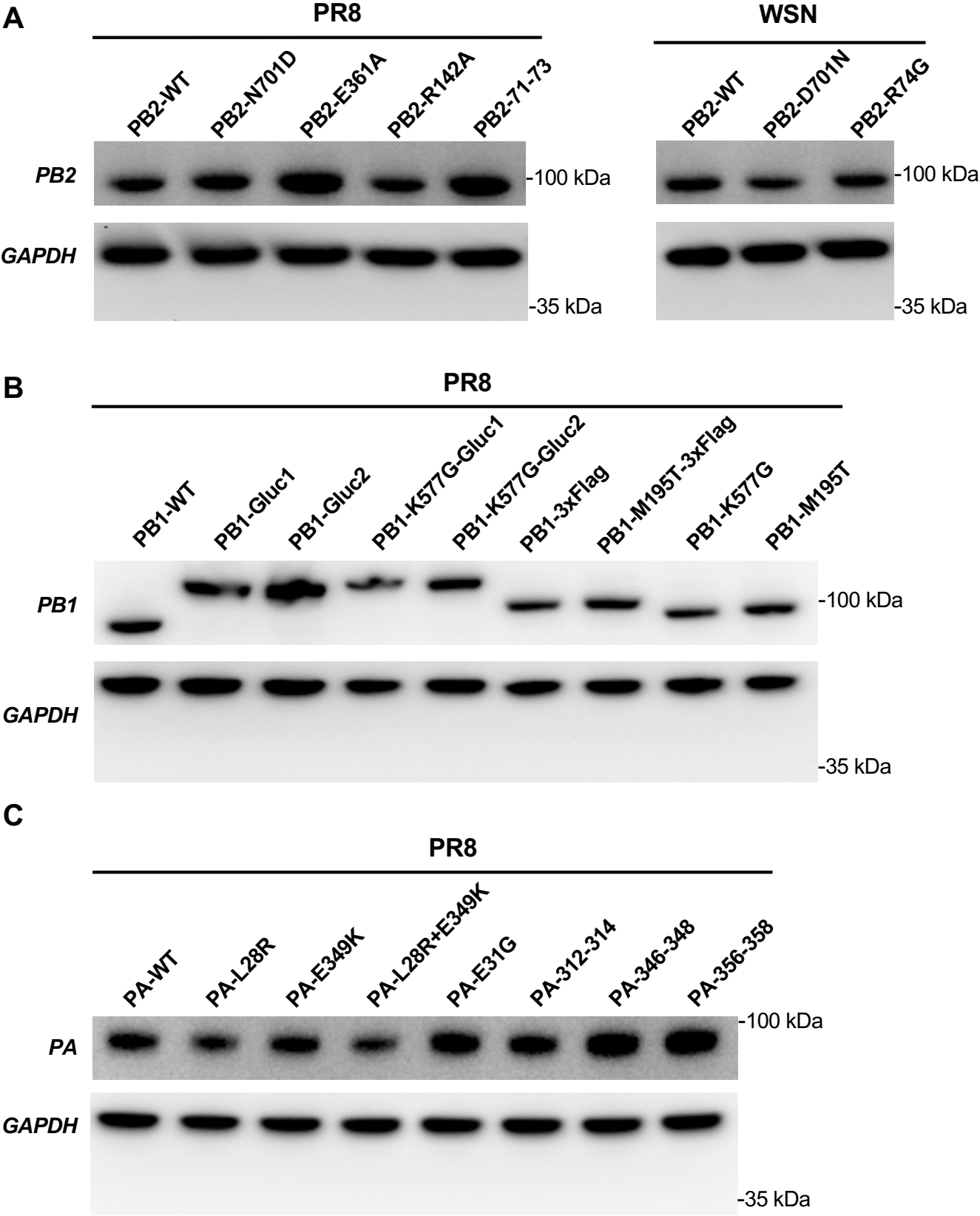

Supplement: S1 Fig — HEK-293T cells were transfected with 50 ng of plasmids expressing the indicated wild-type of mutant FluPol proteins. Total cell lysates were prepared 24 h post-transfection in Laemli buffer and analysed by western blot as described in Diot et al. (Sci Rep 2016, doi: 10.1038/srep33763), using antibodies directed against PB2 (#GTX125925, GeneTex) (A), PB1 (#PA5-34914, Thermo Fisher) (B) and PA (a gift from B. Delmas, INRA Jouy-en-Josas) (C), or a GAPDH loading control antibody (#MA5-15738, Thermo Fisher). Molecular weight markers are indicated. Cropped blots are shown. (PDF) [file ppat.1008034.s001.pdf]

S3 Fig

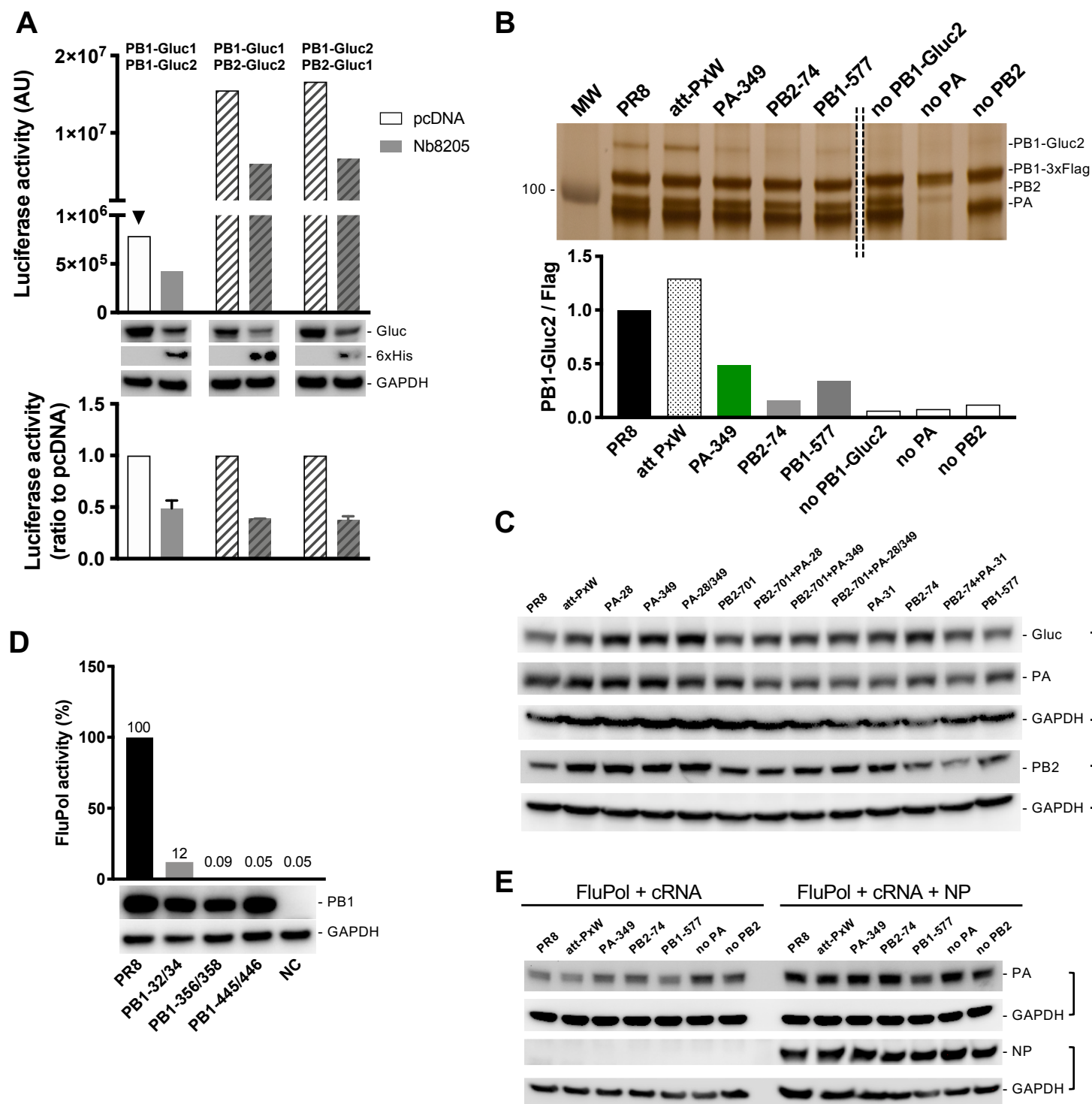

Supplement: S3 Fig — (A) Split luciferase complementation-based assay in an infectious context. Upper panel: HEK-293T cells were transfected with 80 ng of pcDNA3.1 plasmid encoding the nanobody Nb8205 (grey bars) or empty pcDNA 3.1 (white bars) as a control and 24 hours later they were infected at a m.o.i. of 5 with the indicated combinations of recombinant WSN viruses expressing fusion PB1-Gluc1/2 and/or PB2-Gluc1/2 proteins [35] to assess either FluPol dimerization or heterotrimer formation through the PB1-PB1 (open bars) or PB1-PB2 (hatched bars) interactions, respectively. After 6 h of incubation at 37°C, the luciferase enzymatic activity was measured. Western blots to verify expression of the Gluc-tagged PB1/PB2 proteins or 6xHis-tagged nanobody were performed using antibodies directed against Gluc (#E8023S, New England Biolabs) and His-tag (NPB1-41288, Novus Biologicals). The results of one representative experiment (mean of technical triplicates) are shown. Lower panel: the luciferase signals for the PB1-PB1 interaction representative of FluPol dimerization (open bars, mean ± SD of three independent experiments) and for the PB1-PB2 interaction (hatched bars, mean ± SD of two independent experiments), are represented as ratios of signal in the presence of the nanobody Nb8205 (grey bars) over empty pcDNA 3.1 control (white bars). (B) FluPol dimerization assessed by co-immunoprecipitation. HEK-293T cells were co-transfected with plasmids encoding the PR8 polymerase (both PB1-3xFlag and PB1-Gluc2, together with the wild-type PR8-PA and PR8-PB2), the att-PxW polymerase (both PR8-PB1-3xFlag and PR8-PB1-Gluc2 together with PR8-PA and WSN-PB2) or different combinations of the FluPol bearing the reversion mutations in the att-PxW background, as indicated. In the case of the PB1-577 mutation, the K577G mutation was introduced into the PR8-PB1-Gluc2 and PR8-PB1-3xFlag expression plasmids. Controls in the absence of PA or PB2 were also performed. FluPol complexes were purified at 48 h post-t [file ppat.1008034.s003.pdf]

S4 Fig

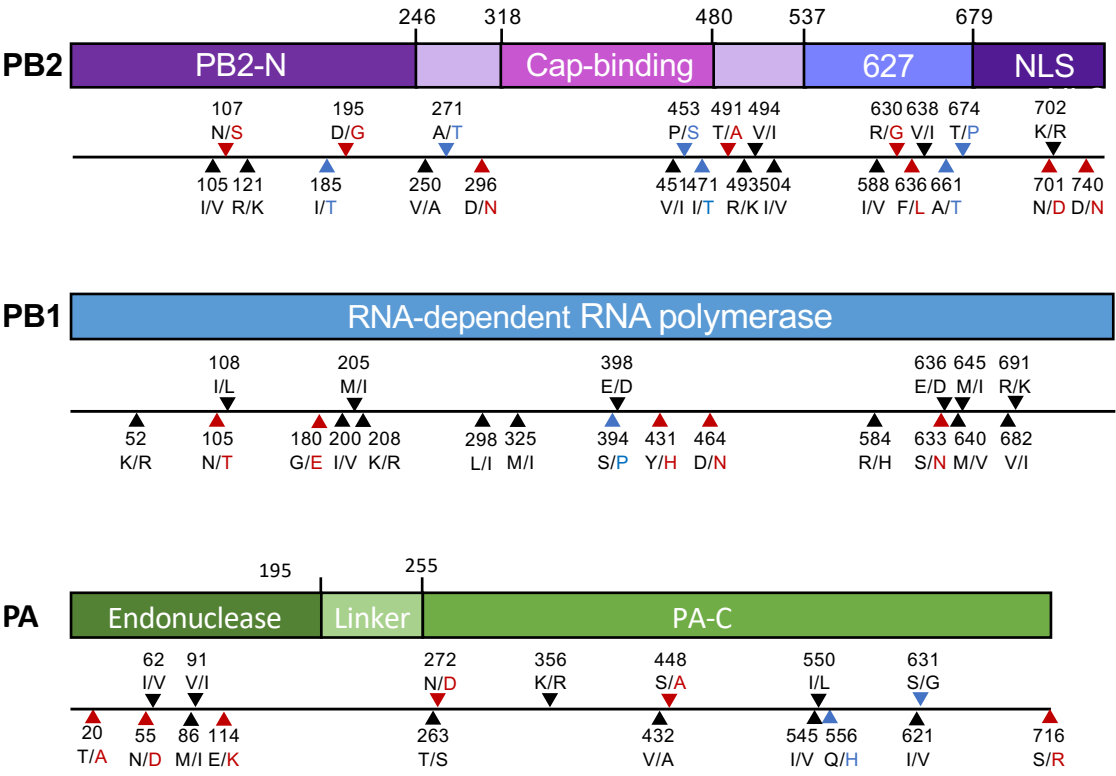

Supplement: S4 Fig — Residues that differ between the PR8 and WSN viral polymerase subunits were mapped on linear representations of the PB2, PB1 and PA protein subdomains (adapted from [4]). The residues that undergo conservative, semi-conservative and non-conservative changes are indicated (amino acid found in PR8/WSN) in black, blue and red, respectively. (PDF) [file ppat.1008034.s004.pdf]
