# Supplementary material for: Influenza virus polymerase subunits co-evolve to ensure proper levels of dimerization of the heterotrimer
Source: PLoS Pathog. 2019 Oct 3;15(10):e1008034. doi: 10.1371/journal.ppat.1008034 (PMC6776259; doi:10.1371/journal.ppat.1008034)

S2 Fig

A

Virus	PB2 74	PA 31	PB1 577	Titer (10^6 PFU/mL)	Plaque phenotype
PR8	G	E	K	200	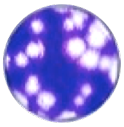
att-PxW	G	E	K	6.5	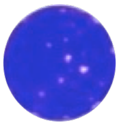
rev-PxW	R	G	G	69	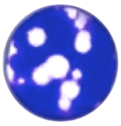
PB2-74	R	E	K	50	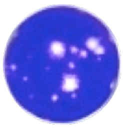
PA-31	G	G	K	3	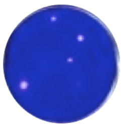
PB2-74+PA-31	R	G	K	5	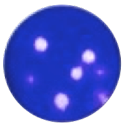
PB1-577	G	E	G	50	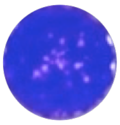

B

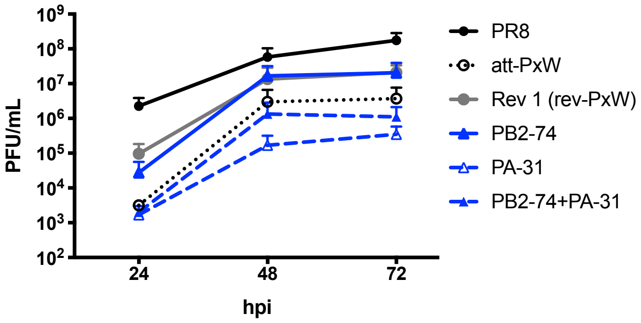

Supplement: S2 Fig — (A) Titers and plaque phenotypes of recombinant viruses bearing one or several reversion mutations in the att-PxW genetic background. The PB2-G74R, PA-E31G, PB2-G74R+PA-E31G, and PB1-K577G mutant viruses were rescued in parallel with the recombinant PR8 and att-PxW viruses. Following one round of amplification on MDCK cells, the titers and plaque phenotypes were compared to that of the rev-PxW virus. (B) Growth kinetics under multi-cycle conditions. A549 cells were infected at a m.o.i. of 0.001 with the indicated viruses. At the indicated times post-infection, viral titers were determined by plaque assay on MDCK cells. The results are shown as the mean ± SD of three independent experiments except for the 72 h time point of Rev 1 that was only measured twice. (PDF) [file ppat.1008034.s002.pdf]
