# Supplementary material for: Influenza virus polymerase subunits co-evolve to ensure proper levels of dimerization of the heterotrimer
Source: PLoS Pathog. 2019 Oct 3;15(10):e1008034. doi: 10.1371/journal.ppat.1008034 (PMC6776259; doi:10.1371/journal.ppat.1008034)

S5 Fig

A

Virus	PB2 701	Titer (10^6 PFU/mL)	Plaque phenotype
PR8	N	200	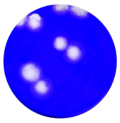
PxW-PB2	D	6.5	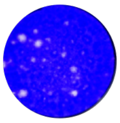
WSN	D	275	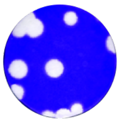
WxP-PB2	N	33	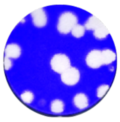

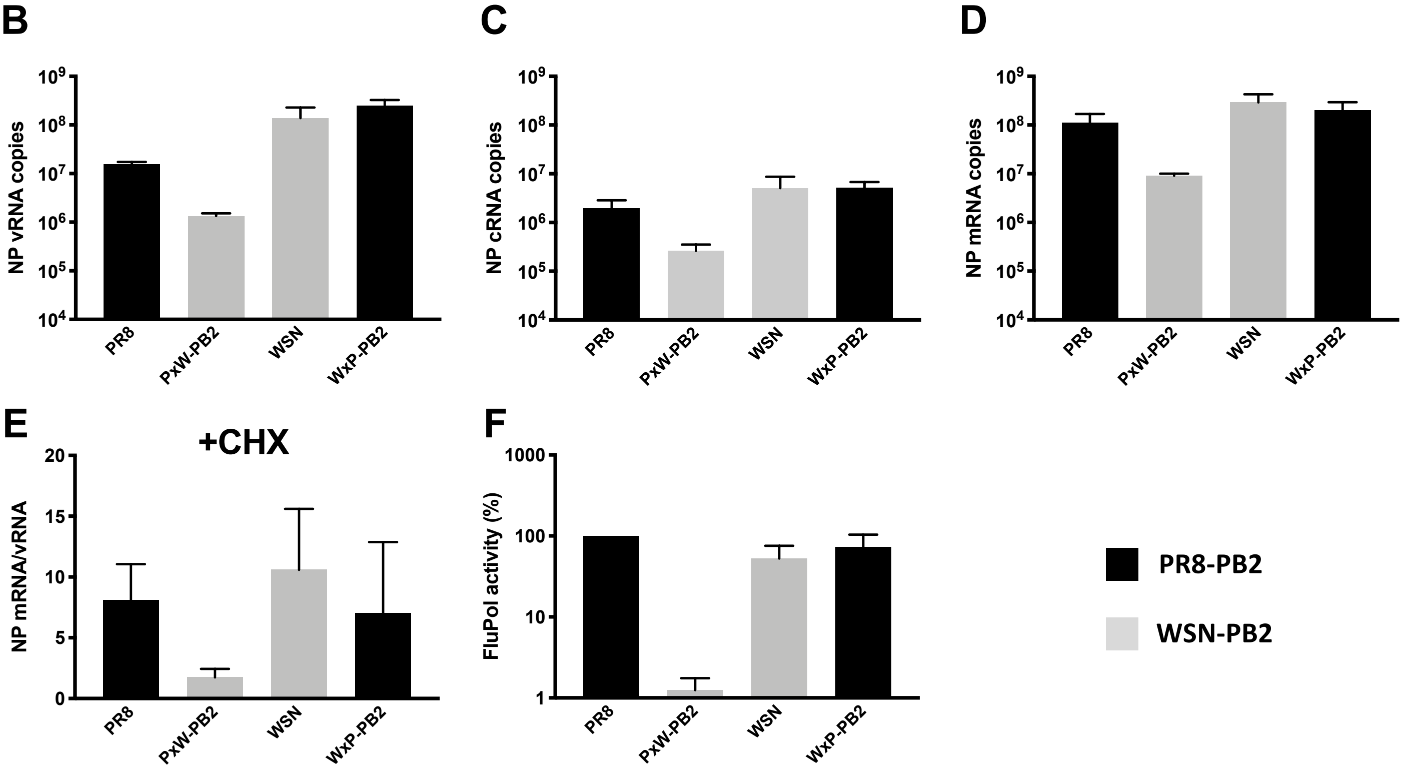

Supplement: S5 Fig — (A) Titers and plaque phenotype of wild-type PR8 and WSN, and reassortant PR8xWSN-PB2 (PxW-PB2) and WSNxPR8-PB2 (WxP-PB2) viruses. The viruses were rescued by reverse genetics and their titers and plaque phenotypes were determined as described in Fig 1. (B-D) Levels of NP v-, c- and mRNAs determined at 6 hpi by strand specific RT-qPCR in A549 cells infected at a m.o.i. of 5. The copy numbers determined as described in Fig 3 are shown as the mean ± SD of three independent experiments in duplicate. (E) Accumulation of primary NP transcripts in the presence of cycloheximide. The mRNA/vRNA ratios are the mean ± SD of 3 independent experiments in duplicates. (F) Activity of the indicated vRNPs as measured in a minigenome assay. The transfections and luciferase read-outs were performed as described in Fig 4A. The results are expressed as percentages (PR8: 100%) and shown as means ± SD of three independent experiments in triplicates. (PDF) [file ppat.1008034.s005.pdf]
