# Supplementary material for: Influenza virus polymerase subunits co-evolve to ensure proper levels of dimerization of the heterotrimer
Source: PLoS Pathog. 2019 Oct 3;15(10):e1008034. doi: 10.1371/journal.ppat.1008034 (PMC6776259; doi:10.1371/journal.ppat.1008034)
Supplement: S1 Table — (PDF) [file ppat.1008034.s006.pdf]

S1 Table. Analysis of sequence variation at each mutation site among a subset of human seasonal IAVs.

Residue	PB2-74	PB2-701	PB1-195	PB1-577	PA-28	PA-349	PA-31
	G: 99.82%	D: 99.25%	M: 90.3%	K: 99.39%	L: 55.11%	E: 99.61%	E: 99.84%
	E: 0.11%	**N: 0.66%**	V: 8.94%	R: 0.5%	P: 44.36%	**K: 0.20%**	X: 0.07%
	**R: 0.02%**	B[b]: 0.07%	I: 0.42%	**N: 0.05%**	S: 0.39%	G: 0.18%	K: 0.05%
	S: 0.02%	E: 0.02%	**T: 0.16%**	M: 0.03%	M: 0.07%		**G: 0.02%**
	X[a]:0.02%		A: 0.08%	**Q: 0.03%**	Q: 0.05%		V: 0.02%
			L: 0.08%		**R: 0.02%**		
			R: 0.03%				
Number of Sequences aligned	4394		3782		4391		

[a] X : not translated when DNA sequence is NNN.
[b] B : translated to D or N when DNA sequence is RAY.
