# Supplementary material for: Influenza virus polymerase subunits co-evolve to ensure proper levels of dimerization of the heterotrimer
Source: PLoS Pathog. 2019 Oct 3;15(10):e1008034. doi: 10.1371/journal.ppat.1008034 (PMC6776259; doi:10.1371/journal.ppat.1008034)
Supplement: S2 Table — (PDF) [file ppat.1008034.s007.pdf]

S2 Table. Mutations detected upon whole genome sequencing of revertant viruses by NGS, present in >10% of total reads, at a given position.

Revertant[a]	Rev 1	Rev 3	Rev 4	Rev 5
**Passage**	P5[b]	P6	P8	P8
**PB2**	D701N (98%)[c]	S286G (81%)<br>P302S (87%)	R251K (67%)<br>R318K (46%)	N153D (38%)<br>C409R (14%)<br>G630R (31%)<br>M645V (13%)
**PB1**	M195T (100%)	K577G (98%)	K577G (100%)<br>I711V (15%)	K577G (100%)
**PA**	L28R (98%)<br>E349K (100%)	L425F (56%)		I129L (14%)<br>L226F (49%)<br>D444S (26%)<br>S631G (14%)
**HA**	K187E (98%)		I230M (37%)	
**NP**				
**NA**		V16A (97%)<br>L22P (95%)	V16A (100%)<br>L22P (100%)	L22P (95%)<br>I26T (61%)
**M**				
**NS**	L27P (99%)			G45R (11%)<br>S17L (26%)

[a] The revertant viruses are numbered according to Fig 5A. For Rev 2, no NGS data are available; Sanger sequencing was used to sequence the PB2, PB1, PA and NP segments, and only the PB2-G74R and PA-E31G mutations were detected.
[b] Px : x serial passages following plaque purification.
[c] In blue : mutations present on a FluPol gene and in ≥ 98% of the reads.
