# Supplementary material for: Influenza virus polymerase subunits co-evolve to ensure proper levels of dimerization of the heterotrimer
Source: PLoS Pathog. 2019 Oct 3;15(10):e1008034. doi: 10.1371/journal.ppat.1008034 (PMC6776259; doi:10.1371/journal.ppat.1008034)
Supplement: S3 Table — (PDF) [file ppat.1008034.s008.pdf]

S3 Table. Primers used in this study.

Primer Name	Sequence (5’ → 3’)
U12	GCCGGAGCTCTGCAGATATCAGCRAAAGCAGG
U12G	GCCGGAGCTCTGCAGATATCAGCGAAAGCAGG
U13	CAGGAAACAGCTATGACAGTAGAAACAAGG
RT-NPvRNA	GGCCGTCATGGTGGCGAATGAATGGACG*A*A*A*AACAAG*A*ATTGC
RT-NPcRNA	GCTAGCTTCAGCTAGGCATCAGTAGAAACAAGGGTATTTTTCTTT
RT-NPmRNA	CCAGATCGTTCGAGTCGTTTTTTTTTTTTTTTTTCTTTAATTGTC
qPCR_Fw-NPvRNA	CTCAATATGAGTGCAGACCGTGCT
qPCR_Fw-NPcRNA	CGATCGTGCC*T*TCCTTTG
qPCR_Fw-NPmRNA	CGATCGTGCC*T*TCCTTTG
Tag_Rv-NPvRNA	GGCCGTCATGGTGGCGAAT
Tag_Rv-NPcRNA	GCTAGCTTCAGCTAGGCATC
Tag_Rv-NPmRNA	CCAGATCGTTCGAGTCGT
